# Caspase 6 deficiency exacerbates inflammatory bowel disease via enterocyte necroptosis and bacterial translocation

**DOI:** 10.1038/s41420-025-02877-z

**Published:** 2025-12-13

**Authors:** Qiong Liu, Jun He, Lixin Liu, Leping Yang, Xiaoyan Qi, Zuxing Wei, Xuyang Hou, Dekun Liu, Yimiao Cheng, Ganglei Liu, Yanwen Zheng, Kuijie Liu

**Affiliations:** 1 Department of Stomatology, The Second Xiangya Hospital, Central South University, Changsha, Hunan China; 2 Department of General Surgery, The Second Xiangya Hospital, Central South University, Changsha, Hunan China

**Keywords:** Gastroenteritis, Necroptosis

## Abstract

Caspase 6 is a pivotal executioner caspase involved in cell death; however, its role in inflammatory bowel disease (IBD) remains incompletely understood. Levels of cleaved caspase 6 were quantified in colonic tissues from IBD patients, and an IBD mouse model was established via DSS induction, incorporating both systemic (*Casp6* KO) and IEC-specific knockout (*Casp6* cKO) strategies. Single-cell RNA sequencing (scRNA-seq) revealed that *Casp6* KO enhanced necroptosis in IECs, reducing intestinal endocrine cells and damaging intestinal stem cells. Both in vivo and in vitro studies confirmed that caspase 6 deficiency activates the necroptosis pathway by upregulating RIPK1 in IECs and impairs macrophage bacterial clearance. Importantly, *Casp6* KO reduces bactericidal activity in a cathepsin L (CTSL)-dependent manner. These findings demonstrate that preserving caspase 6 activity is essential for necroptosis prevention and effective bacterial clearance, providing new insights for future IBD therapies.

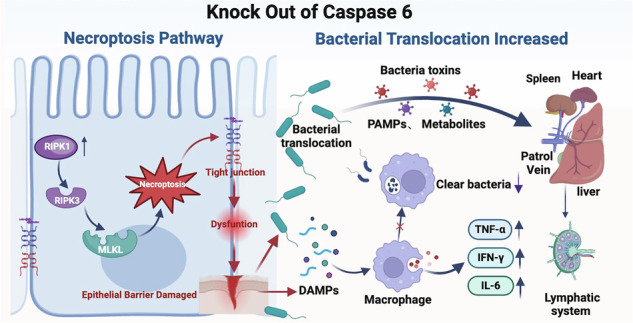

## Introduction

Inflammatory bowel diseases (IBDs), including Crohn’s disease and ulcerative colitis, a chronic and recurrent disease characterized by intestinal inflammation and epithelial damage [1]. In recent decades, there has been a significant increase in the incidence of IBDs in both developing and developed countries, leading to a greater global burden of disease [2]. While the exact pathogenesis of IBDs remains unclear, relevant studies have shown that the disruption of the epithelial barrier function plays a crucial role in the development of IBDs [3, 4]. The adhesion of intestinal epithelial cells (IECs) is responsible for the generation of intestinal barrier function [5]. Excessive cell death of IECs compromises the integrity of the intestinal barrier, facilitating the invasion of luminal antigens into the lamina propria and resulting in chronic inflammation [6]. In this context, previous studies have suggested an elevated occurrence of apoptotic epithelial cell death among patients with IBDs [7–9]. As the research moves along, the increasing body of research has demonstrated that necroptosis, an alternative form of cell death, can take place in epithelial cells during the inflammatory progression of IBDs [10–12].

Cell death resulting from various injuries primarily presents as apoptosis and necrosis. Apoptosis primarily eliminates cells in a non-inflammatory manner. During this process, caspase family members, which are cysteine proteases, are sequentially activated, leading to the breakdown of cells into membrane-wrapped fragments known as apoptotic bodies that are rapidly engulfed by resident macrophages or neighboring cells [13–15]. Apoptosis spontaneously occurs in IECs and maintains a delicate balance with epithelial cell proliferation, ensuring the maintenance of normal intestinal morphology and function [16]. However, under conditions of local or systemic inflammation, the disruption of apoptosis and epithelial cell proliferation balance can result in the pathological shedding of IECs [17]. Necroptosis is an accidental form of cell death characterized by significant cellular damage and the subsequent release of intracellular components that trigger the activation of innate immune cells [18]. This process is accomplished through the sequential activation of receptor-interacting protein kinases (RIPK) 1, RIPK3, and downstream effectors [19]. Recent studies have demonstrated a significant association between necroptosis and intestinal inflammation in both mice and children with IBDs [20, 21]. The mortality and tissue damage observed in mice with necroptotic IECs were significantly more severe compared to apoptosis [4].

The activity of caspases plays a crucial role in preventing necroptosis in IECs [4, 22]. Caspases, a family of cysteine proteases, are responsible for cleaving substrates after aspartic acid residues and play a significant role in apoptosis, necrosis, and inflammation [22]. While the role of caspases in regulating apoptosis has been extensively studied, the complexity of their involvement in the transition between apoptosis and necroptosis is still unexpected. Caspase inhibition prevented apoptosis in certain cells but ultimately resulted in necrotic cell death. The main cause of this transition was identified as the inhibition of caspase 8, which subsequently led to the activation of the RIPK pathway [23–25]. The activation of caspase 8 is typically associated with increased apoptosis and shedding of IECs in studies on the caspase family and intestinal homeostasis. However, the deletion of caspase 8 in epithelial cells can result in necroptosis and exacerbate intestinal injury [4]. Similarly, caspase 1 also plays a crucial role in repairing damaged tissues in the model of dextran sulfate sodium (DSS)-induced IBDs. Nevertheless, in the absence of caspase 1, mice exhibited increased colonic inflammation compared to the control group [26]. Thus, the activity of caspase is closely associated with the prognostic outcomes of gastrointestinal diseases.

There is vivid activity exploring the role of caspases in intestinal homeostasis, such as caspase 8. However, the role of caspase 6 in IBDs has received limited attention. Studies have demonstrated that caspase 6 plays a critical role in regulating innate immunity, activating inflammasomes, and enhancing host defense mechanisms [27]. To gain insight into the biological function of caspase 6 in intestinal epithelial injury, we investigated the expression of caspase 6 in the colon tissues of patients with IBDs and healthy individuals. Our findings revealed a positive correlation between caspase 6 expression and the severity of IBDs. Surprisingly, we observed that caspase 6-knockout (*Casp6* KO) exacerbated DSS-induced IBDs in mice. To elucidate the underlying mechanism through which caspase 6 depletion worsens IBDs, we employed single-cell RNA sequencing (scRNA-seq) to analyze the colon tissues of *Casp6* KO mice and their respective controls, aiming to identify the key factors involved in the progression of IBDs. Extensive necrosis was observed in various IECs in individuals with IBDs. Moreover, our study confirmed that inhibiting caspase 6 activity worsens the progression of IBD by inducing necroptosis in IECs and enhancing intestinal bacterial translocation. These findings thus improve our understanding of the mechanisms driving IBD progression and may yield novel prognostic markers for its advanced stages.

## Results

### The severity of IBDs was exacerbated by systemic *Casp6* KO

**Fig. 1:**
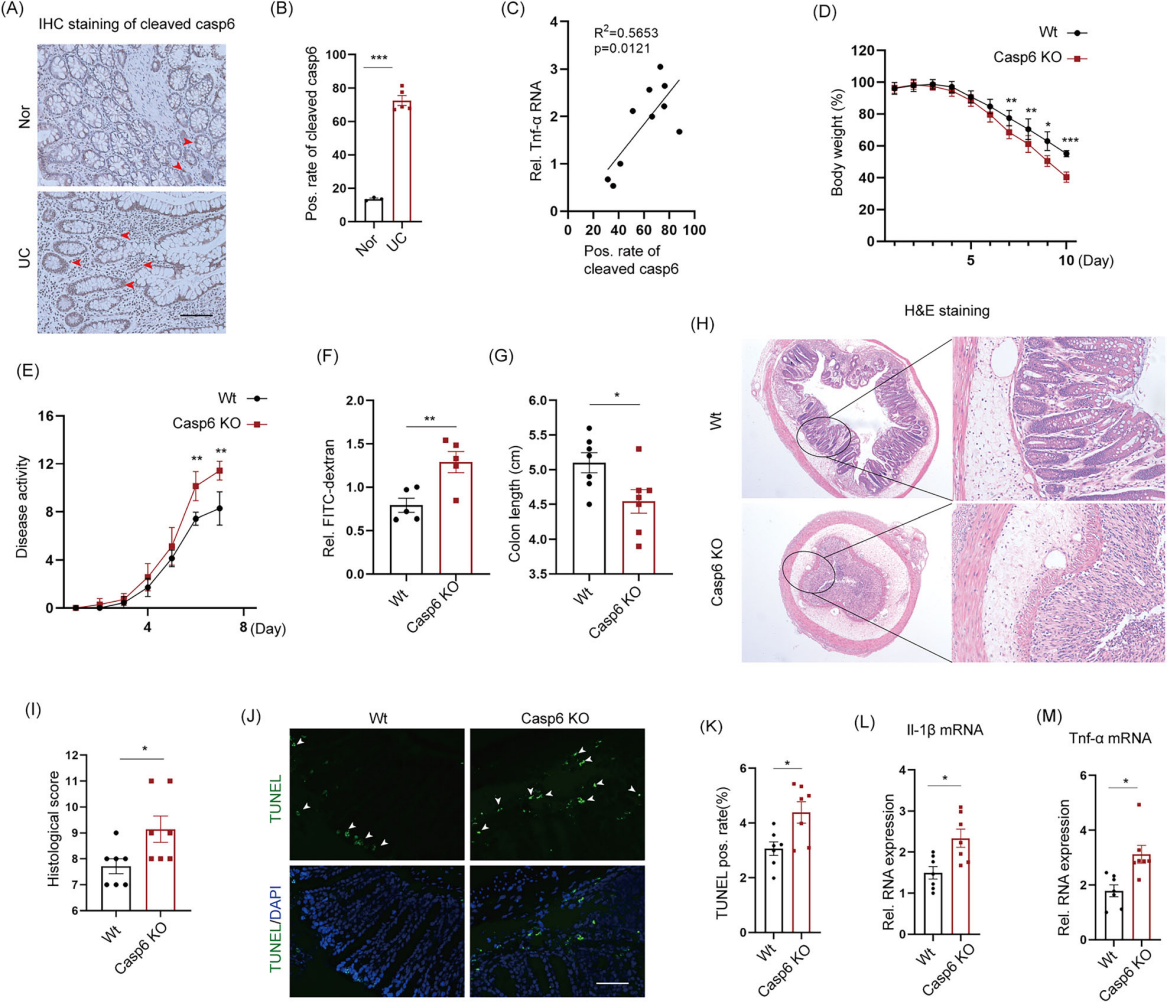
The severity of IBD was exacerbated by systemic *Casp6* KO. **A** Representative images of IHC staining for cleaved caspase 6 (casp6) in colon tissues of both normal individuals and patients with ulcerative colitis (UC). Positive staining is indicated by brown coloration (arrows). Scale bar = 50 μm. **B** Quantification of IHC staining shows the percentage of positive cells in each group (*n* = 3–5). **C** The expression level of cleaved casp6 was examined using IHC in 10 patients, while the level of Tnf-α RNA was measured using qPCR. The correlation between the expression of cleaved casp6 and Tnf-α was analyzed, yielding an *R*^*2*^ value of 0.5653 and a *P* value of 0.0121. **D** The IBD model was induced by administering 2.5% DSS, and the changes in body weight were compared between Wt and *Casp6* KO mice. **E** DAI scores were recorded for Wt and *Casp6* KO mice. **F** Intestinal permeability assessed by administering FITC-dextran via the intragastric route (*n* = 5). **G** Colon length was compared between Wt and *Casp6* KO mice (*n* = 7). **H**, **I** H&E staining and histological analysis were performed on the colon tissue sections of Wt and *Casp6* KO mice (*n* = 7). **J**, **K** Representative images and quantitative analysis of TUNEL staining for colon tissue in each group (*n* = 7). Scale bar = 50 μm. **L**, **M** Q-PCR detection of Tnf-α and Il-1β in the colons of Wt and *Casp6* KO mice (*n* = 7). * *P* < 0.05, ** *P* < 0.01 and *** *P* < 0.001.

To investigate the potential functional significance of caspase 6 in IBD, we analyzed changes in the expression levels of cleaved caspase 6 in the colonic tissues of patients with IBD. Immunohistochemical (IHC) analysis revealed that the expression of cleaved caspase 6 was significantly elevated in IBD patients compared to the control group (Fig. 1A, B). To further validate the upregulation of caspase 6 activity in IBD, we performed a correlation analysis between the cleaved caspase 6 positive rate (determined by IHC staining) and Tnf-α mRNA expression levels (measured by qPCR) in intestinal tissues from 10 clinical patients. The analysis showed a moderate positive correlation with an *R*^*2*^ value of 0.5653 and a statistically significant *P* value of 0.0121, indicating that higher cleaved caspase 6 activity is associated with increased Tnf-α expression in IBD tissues (Fig. 1C and S1A, B). This finding suggests a positive correlation between the expression of caspase 6 and the severity of IBDs. Therefore, we generated *Casp6* KO mice and utilized a 2.5% DSS-induced IBDs model to investigate the pathological impact of caspase 6 activity in IBDs (Fig. S1C–E). During IBD model induction, we observed that caspase 6-deficient mice displayed more significant weight loss, higher DAI scores, increased intestinal permeability, and a greater reduction in colon length over time compared to wild-type (Wt) mice (Fig. 1D–G and Fig. S1F). After Hematoxylin & eosin (H&E) staining of the colonic tissues, the gene knockout group showed marked disruption of the intestinal mucosal structure, irregular arrangement of the intestinal glands, reduced or even absent crypts, significant edema of the lamina propria with extensive infiltration of inflammatory cells, and epithelial shedding in some areas; whereas these pathological changes were much less pronounced in the Wt group compared to the caspase 6 knockout group (Fig. 1H). Pathological scoring indicated that the gene knockout group had a significantly higher intestinal injury score compared to the control group (Fig. 1I). Additionally, TUNEL staining and quantitative real-time PCR (qPCR) analysis revealed that *Casp6* KO mice exhibited a notable increase in both cell apoptosis and the expression of pro-inflammatory cytokines, including Tnf-α and Il-1β mRNA, in the colon affected by IBDs (Fig. 1J–M).

### The results of scRNA-seq indicated that the knockout of caspase 6 promoted necroptosis in IECs

**Fig. 2:**
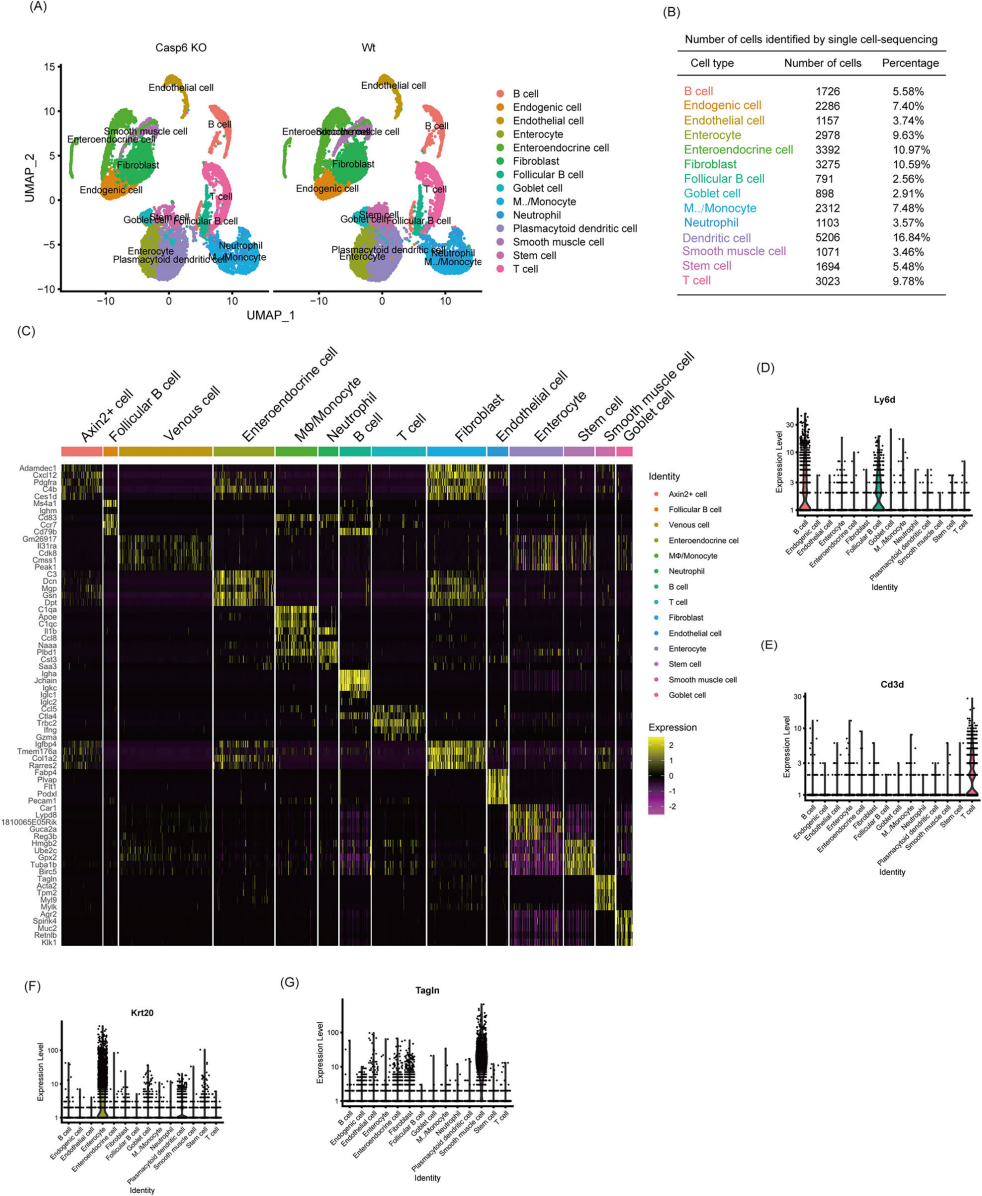
ScRNA-seq was employed to analyze the transcriptome of colon tissue from *Casp6* KO and Wt mice after inducing IBD modeling. **A** By employing the UMAP method for dimensionality reduction analysis, it demonstrates the main cell types in the colonic IBD. **B** The number and percentage of cells for each cell type are shown. **C** The heatmap displays the expression levels of specific markers in each cell type. **D**–**G** Violin plots show the expression of representative and well-known markers in different cell types identified in the colonic IBD. The *y*-axis represents the normalized read count.

We conducted scRNA-seq analysis on the colons of Wt and *Casp6* KO mice to investigate the unexpected exacerbation of IBD progression caused by caspase 6 deletion (Fig. S2A). After applying multiple layers of quality control, we obtained a total of 30,912 single-cell transcriptomic data, consisting of 17,922 cells from Wt mice and 12,990 cells from *Casp6* KO mice. By performing principal component analysis on all cells, we identified 14 distinct cell types (Fig. 2A). We employed differential gene expression analysis to identify the characteristic marker genes for each cell type. The top five cell types were identified as dendritic cells, enteroendocrine cells, fibroblasts, T cells, and enterocytes (Fig. 2B). The expression of common cell marker genes was observed in all cell types (Fig. 2C–G). For goblet cells, the genes Muc2 and Agr2 were expressed, while enterocyte cells expressed Reg3b and Fabp4. T cells showed expression of Cd3d, Ifng, and Trbc2, while B cells expressed Igha, Ly6d, and Iglc1. Fibroblasts expressed Col1a2 and Col3a1, enteroendocrine cells expressed Dcn and Mgp, endothelial cells expressed Plvap and Pecam1, enterocytes expressed Krt20, and smooth muscle cells expressed Tagln.

**Fig. 3:**
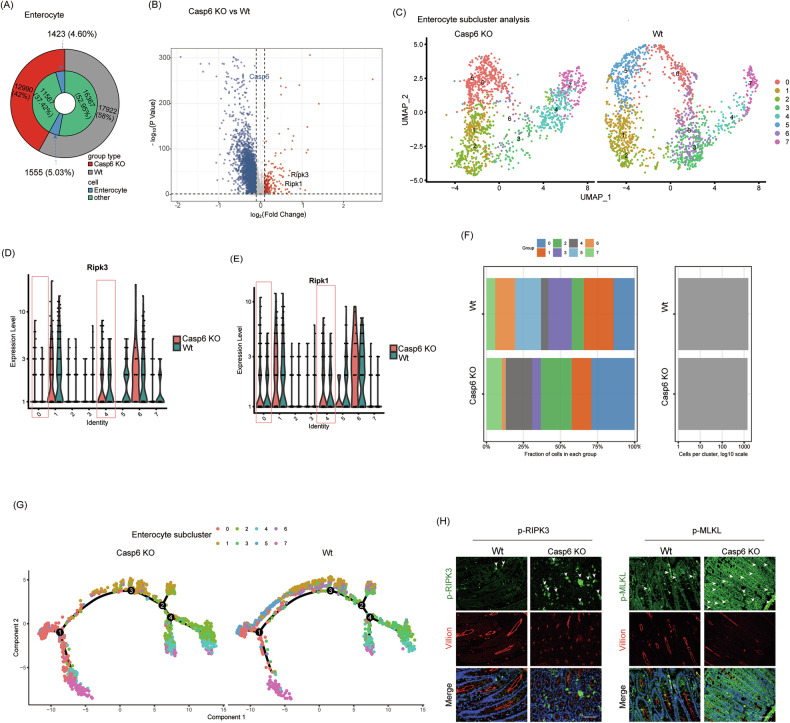
ScRNA-seq showed that *Casp6* KO promoted necroptosis in IECs. **A** Comparison of the IEC count between *Casp6* KO and Wt mice. **B** Differential gene expression in enterocytes between *Casp6* KO and Wt mice, with a statistical significance of (|log_2_(fold change)å 0.5, and *P* < 0.05|). **C** The dimensionality reduction analysis was performed by UMAP, and 8 subgroups were obtained from sub-clustering of IECs in IBD. **D**, **E** Expression patterns of Ripk1 and Ripk3 in 8 subpopulations. **F** Proportion of each subpopulation of cells in *Casp6* KO and Wt mice. **G** Trajectory analysis was employed to trace the differentiation trajectory of IECs. **H** Immunofluorescence analysis was conducted on the intestines of *Casp6* KO and Wt mice to examine the expression of necroptosis activity marker proteins, including p-RIPK3 and p-MLKL, in IECs (*n* = 7). Scale bar = 50 μm.

To investigate the mechanism by which *Casp6* KO exacerbates IBDs, we conducted further analysis of the single-cell data. Considering that enterocyte loss is an early hallmark of IBDs, we examined the enterocytes and observed a reduction in the absolute number of IECs following *Casp6* KO (Fig. 3A). However, it is important to note that this reduction in cell number is proportional to the overall decrease in cell yield from the *Casp6* KO samples, and the relative proportion of enterocytes among total cells remains comparable between *Casp6* KO and Wt groups. Therefore, the biological significance may lie less in the absolute number of enterocytes captured and more in the changes in enterocyte subcluster distribution and cell state composition. A comparison of gene expression between *Casp6* KO and Wt enterocytes revealed 625 differentially expressed genes with a cutoff of (|log_2_(fold change) >0.5, and *P* < 0.05 | ) (Fig. 3B and Table S1). In the *Casp6* KO group, the expression of key genes involved in necroptosis, such as Ripk1 and Ripk3, increased, while the expression of key genes related to apoptosis and autophagy, such as LC3, p62, caspase 3, and BAX, showed no significant difference. To gain a better understanding of the heterogeneity of IECs following IBDs modeling, we conducted dimensionality reduction analysis, which resulted in the identification of 8 distinct subgroups (Fig. 3C and Table S2). Among these subpopulations, subclusters 6 and 1 exhibit markedly distinct distributions and gene expression patterns. Specifically, subcluster 6 is significantly enriched in the *Casp6* KO group, with a notable increase in Ripk1 and Ripk3 expression compared to the Wt; In contrast, subcluster 1 does not exhibit a significant upregulation of these necroptosis markers, and its distribution is minimally affected by the caspase 6 status (Fig. 3D, E). Furthermore, subclusters 5 and 6 exhibit substantial differences in Ripk1 and Ripk3 expression patterns; subcluster 6 consistently shows higher expression of both genes under *Casp6* KO conditions, whereas subcluster 5 maintains relatively lower levels, indicating functional heterogeneity among these cellular states (Fig. 3D, E). The total number of cells in necroptosis-prone subclusters 0 and 4 increased from 19.1% in the control group to 46.9% in the *Casp6* KO group (Fig. 3F), further highlighting *Casp6* KO’s impact on enterocyte fate. Trajectory analysis of all enterocytes revealed a continuum from a necroptosis-free starting point to terminal states characterized by high necroptosis gene expression (Fig. 3G). Notably, subclusters 5 and 6 followed distinct differentiation trajectories, with subcluster 6 occupying the terminal, necroptosis-associated state, while subcluster 5 resided along an alternative path with low necroptosis marker expression (Fig. 3G). To verify the induction of necroptosis in IECs by *Casp6* KO, immunofluorescence analysis was performed on the intestines of *Casp6* KO and Wt mice to detect the expression of necroptosis activity marker proteins such as p-RIPK3 and p-MLKL in IECs. Immunofluorescence staining showed that p-RIPK3 and p-MLKL protein levels were low in the colonic tissues of the control group, with weak and punctate fluorescence signals (Fig. 3H). In contrast, the gene knockout group exhibited markedly elevated expression of the target proteins, with much stronger fluorescence signals and patchy regions of high expression (Fig. 3H). These results indicate that *Casp6* KO does not simply reduce the absolute number of enterocytes, but rather reshapes the distribution and fate of enterocyte subpopulations, driving a shift toward necroptosis-prone cell states. This abnormal activation of necroptosis is likely a primary factor contributing to the exacerbation of IBDs in the *Casp6* KO context. Further in-depth analysis of the functional heterogeneity among enterocyte subclusters and the molecular mechanisms involved will be conducted in future studies.

**Fig. 4:**
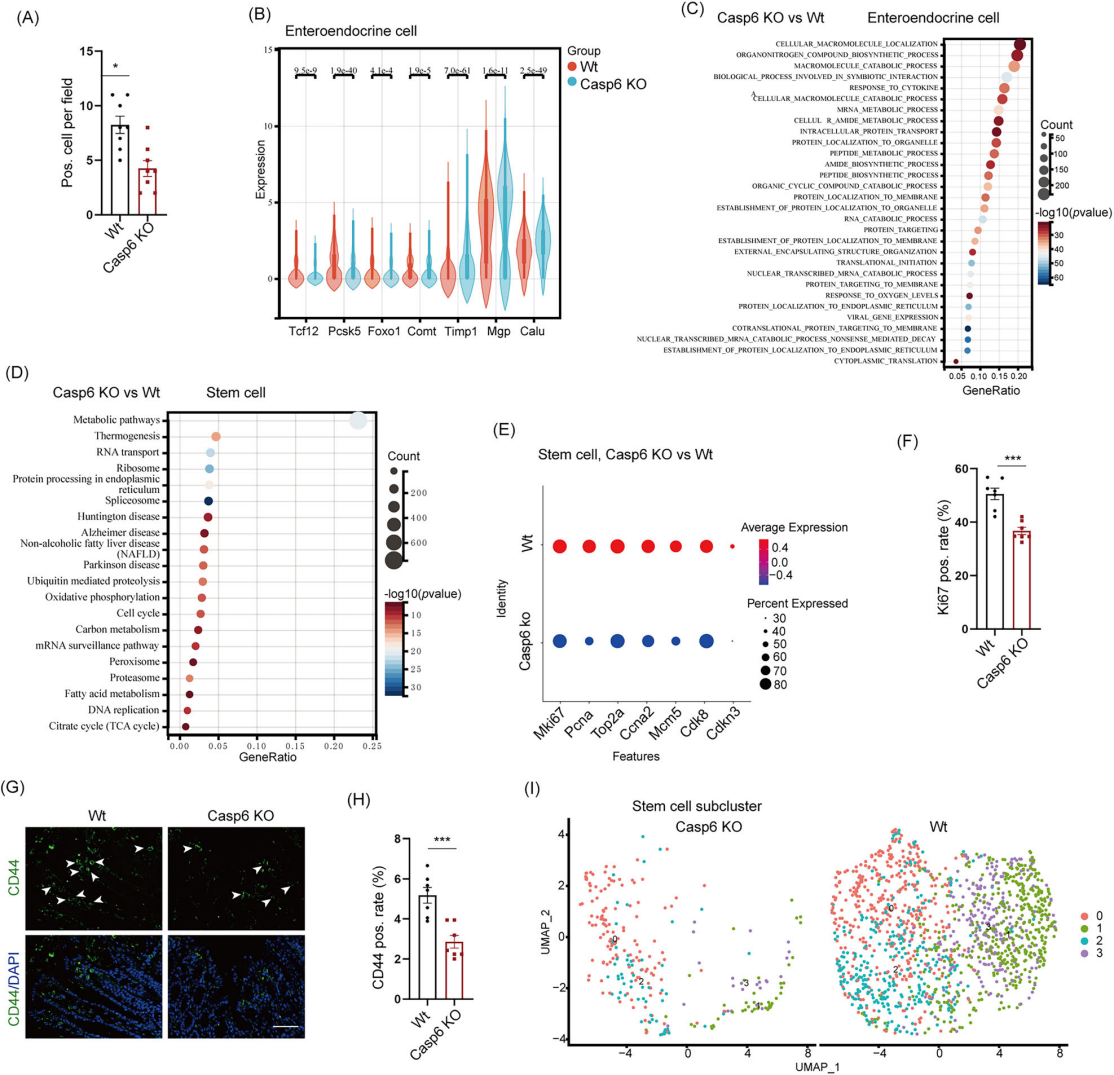
The results of scRNA-seq indicated that *Casp6* KO led to a decrease in the population of enteroendocrine cells and impairment of intestinal stem cells. **A** The quantitative results of IHC staining for Chromogranin A (CGA) in enteroendocrine cells showed the percentage of positive cells in each group (*n* = 8). **B** Violin plots were generated to illustrate significant changes in the expression of regulatory genes associated with enteroendocrine cells between *Casp6* KO and Wt. **C** Functional enrichment analysis was performed on the enteroendocrine cells. **D** KEGG analysis was conducted to analyze the differentially expressed genes in stem cells. **E** The differential expression of essential genes related to proliferation and differentiation was investigated. **F** The quantitative results of IHC staining for Ki67 in enteroendocrine cells showed the percentage of positive cells in each group (*n* = 7). **G**, **H** The stem cell marker CD44 in colonic tissue was assessed using IHC staining and quantitative analysis (*n* = 7). Scale bar = 50 μm. **I** By employing the UMAP method for dimensionality reduction analysis, 4 distinct subpopulations of stem cells were identified. * *P* < 0.05, *** *P* < 0.001.

Additionally, our findings indicate a significant reduction in the number of enteroendocrine cells in the *Casp6* KO group, with a decrease from 6.9% to 4.0% (Fig. S3A). Furthermore, IHC staining revealed that the expression of Chromogranin A, a marker protein for enteroendocrine cells, was low in the colonic tissues of the gene knockout group, as evidenced by weak positive signals and light brownish-yellow staining (Fig. S3B). In contrast, the Wt group exhibited significantly higher expression levels of the target protein, with an expanded area of positive staining and more pronounced brownish–yellow signals (Fig. S3B). Semi-quantitative analysis demonstrated that the average proportion of target protein-positive cells in the Wt group was significantly greater than in the gene knockout group (Fig. 4A). Notably, significant changes have been observed in enteroendocrine secretory products, including Chromogranin A, PYY, CCK, GLP-1, 5-HT, somatostatin, gastrin, and motilin, throughout the progression of IBDs. The crucial roles played by enteroendocrine cells in the pathogenesis and symptomatology of IBDs have been widely acknowledged [28]. In the *Casp6* KO mice, the expression of Tcf12 and Calu, which regulate enteroendocrine cell differentiation, as well as Pcsk5, which regulates PYY secretion, and Foxo1, a transcription factor for CCK transcription, were downregulated (Fig. 4B). Conversely, the expression levels of Comt, which inhibits 5-HT activity, and other important regulatory genes such as Timp1 and Mgp, were upregulated (Fig. 4B). Functional enrichment analysis of enteroendocrine cells revealed that *Casp6* KO led to a significant enrichment of pathways related to protein synthesis and secretion, including peptide biosynthesis, protein processing in the endoplasmic reticulum, and membrane localization (Fig. 4C and Table S3). Additionally, there was an enrichment of pathways related to stimulus response, such as cytokine or oxygen level response (Fig. 4C and Table S3). The UMAP method was employed to perform dimension reduction analysis on enteroendocrine cells, revealing the presence of 7 distinct enteroendocrine subsets (Fig. S3C and Table S4). Among these subsets, subcluster 0 exhibited the most significant decrease in cell population following *Casp6* KO (Fig. S3D). The KEGG analysis of subcluster 0 and other subclusters indicated that subcluster 0 was the primary subpopulation of enteroendocrine cells that responded to external or endogenous stimuli (Fig. S3E). These findings suggest that the dysregulation in the quantity and function of enteroendocrine cells could potentially contribute to the exacerbation of IBDs following *Casp6* KO. However, the precise underlying mechanisms require further investigation.

The gut possesses the ability to undergo self-renewal, while stem cells can differentiate into various cell types, including intestinal epithelial and endothelial cells. This differentiation capacity indicates that stem cells may play a role in tissue regeneration and repair in the inflamed gut [29]. Consequently, an inadequate number or functional loss of stem cells can result in the progression of IBDs. Our scRNA-seq data revealed a decrease in the number of stem cells from 4.6% in the control group to 0.8% in the *Casp6* KO group (Fig. S3F). KEGG analysis of differentially expressed genes related to stem cells demonstrated a significant correlation between *Casp6* KO and pathways associated with stem cell proliferation, including the cell cycle and DNA replication (Fig. 4D). Subsequent analysis revealed a significant reduction in the expression of crucial markers associated with the proliferation and differentiation of intestinal stem cells, such as Mki67, Top2a, and Cdk8, following *Casp6* KO (Fig. 4E). Ki67 expression in the colon was detected using IHC revealing low expression in *Casp6* KO mice (Figs. S3G and 4F). Immunofluorescence staining demonstrated significantly lower expression of the stem cell marker CD44 in *Casp6* KO mice compared to Wt mice (Fig. 4G, H). Dimensionality reduction analysis of intestinal stem cells identified four distinct cell subgroups (Fig. 4I and Table S5), among which subcluster 1 exhibited the most pronounced reduction following *Casp6* KO (Fig. S3H). These results suggest that the abnormal quantity of intestinal stem cells and the functionality of proliferative renewal cells could potentially contribute to the exacerbation of IBDs following *Casp6* KO. However, further investigation is required to elucidate the underlying mechanism.

### Targeted deletion of caspase 6 in IECs also exacerbates IBDs

**Fig. 5:**
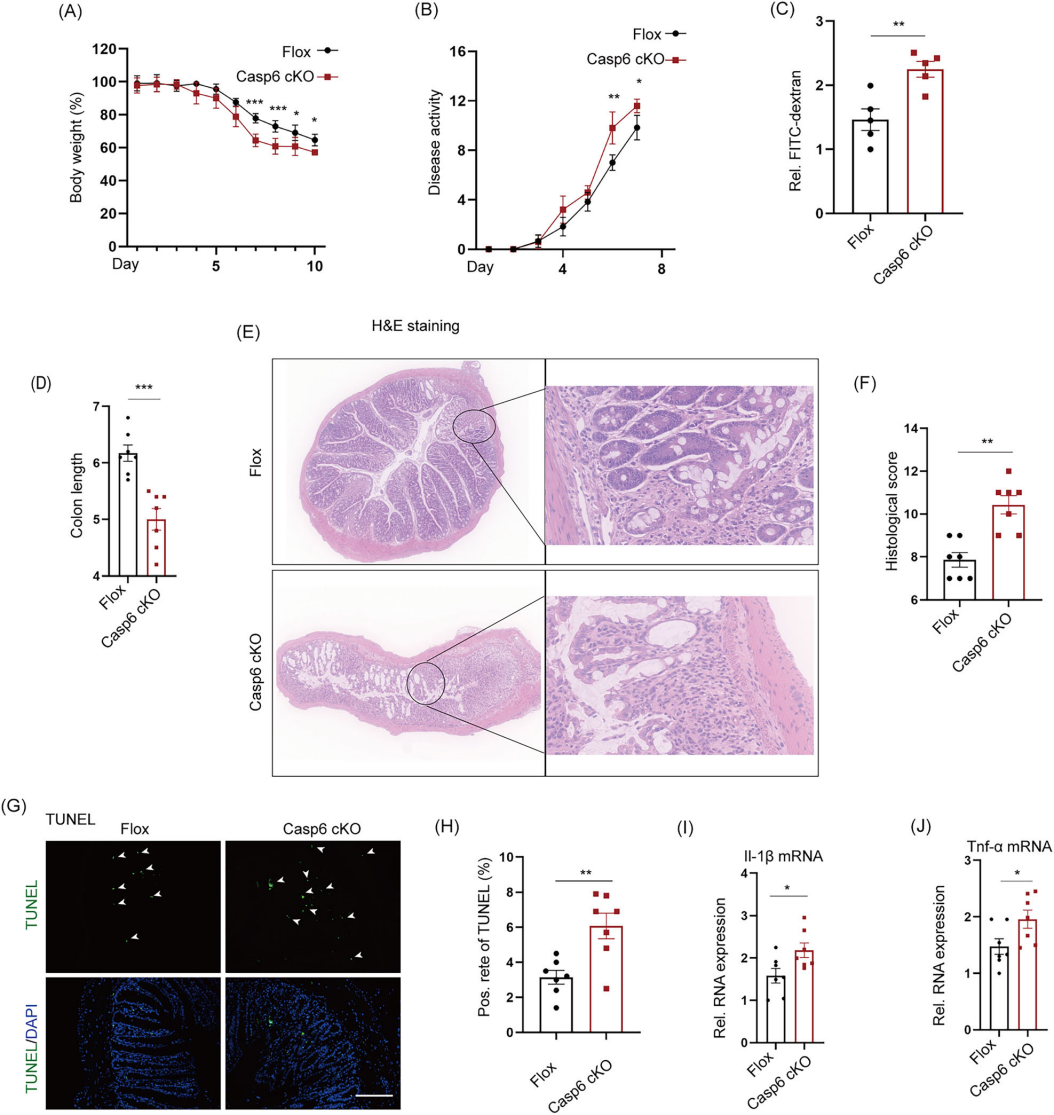
Knockout of caspase 6 specifically in IECs also exacerbates IBD. **A** Comparison of body weight between Flox and cKO mice after inducing IBD using 2.5% DSS. **B** DAI scores of Flox and cKO mice. **C** Intestinal permeability was assessed through intragastric administration of FITC-dextran (*n* = 5). **D** Comparison of colon length between Flox and cKO mice (*n* = 7). **E**, **F** H&E staining and histological analysis of the colon in Flox and cKO mice (*n* = 7). **G**, **H** TUNEL assay and quantitative analysis of were performed to analyze colon tissue cell apoptosis in the two groups (*n* = 7). Scale bar = 50 μm. **I**, **J** Q-PCR detection of Tnf-α and Il-1β in the colons of Flox and cKO mice (*n* = 7). * *P* < 0.05, ** *P* < 0.01, and *** *P* < 0.001.

The ScRNA-seq results strongly suggest a potential association between the aggravation of IBDs and necroptosis of IECs caused by *Casp6* KO. In order to assess the validity of this hypothesis, we created mice with a specific knockout of caspase 6 in IECs (*Casp6* cKO). Figure S4A illustrates the construction strategy for generating caspase 6 Flox mice, Fig. S4B presents the identification strategy for Flox and cKO mice, and Fig. S4C, D provide a schematic diagram and weight measurements of 6-week-old mice, demonstrating that *Casp6* cKO does not impact mouse development. Six-week-old Flox and cKO mice were subjected to a 2.5% DSS-induced model, and samples were collected after 7 days. The results indicated that cKO mice experienced greater weight loss compared to Flox mice (Fig. 5A). The disease activity score was recorded for both Flox and cKO mice, revealing a significant aggravation in *Casp6* cKO mice (Fig. 5B). In addition, cKO mice exhibited increased intestinal permeability and a notable reduction in colon length (Figs. 5C, D and S4E). H&E staining of the colon tissue demonstrated an exacerbation in the destruction of mucosal integrity and a significant increase in pathological damage in the colon of *Casp6* cKO mice (Fig. 5E). Furthermore, we conducted TUNEL staining and qPCR experiments, which revealed that *Casp6* cKO not only enhanced colon cell death but also upregulated the expression of inflammatory cytokines, including Il-1β and Tnf-α (Fig. 5G–J). The severity of IBDs was further aggravated by the specific *Casp6* cKO, consistent with the results of caspase 6 systemic knockout. These results indicate that the deficiency of caspase 6 exacerbates the severity of IBDs in a mouse model.

### The deficiency of caspase 6 in IECs enhances necroptosis through upregulation of RIPK1 protein expression

**Fig. 6:**
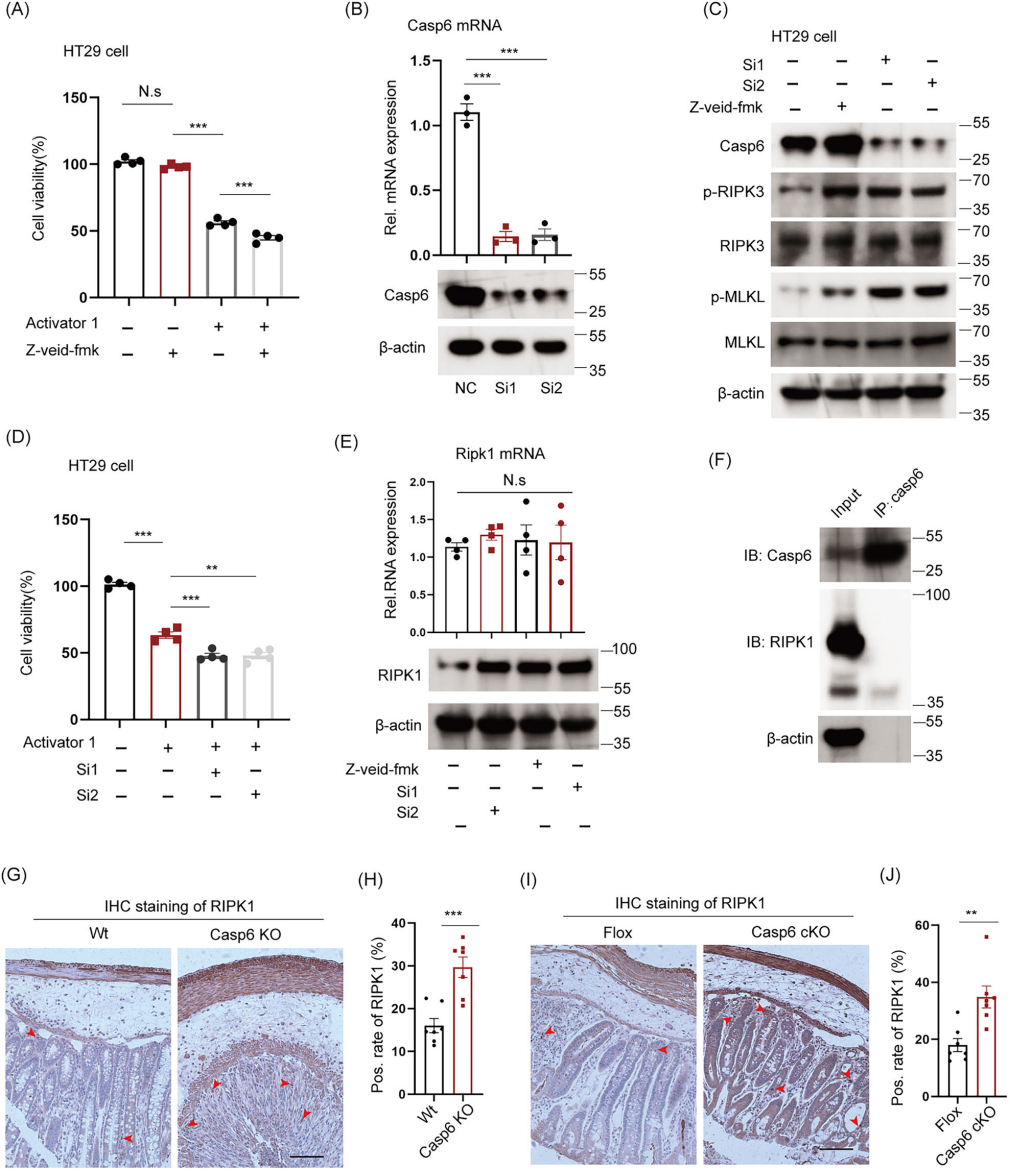
*Casp6* KO in IECs enhances necroptosis by upregulating the protein expression of RIPK1. **A** HT29 cells were treated with RIPK1/RIPK3/MLKL Activator 1 and/or caspase 6 inhibitors (Z-veid-fmk), and the cell viability was assessed using CCK8 assay (*n* = 4). **B** Caspase 6 was knocked down in HT29 cells using siRNA transfection, and the expression level of caspase 6 was assessed by qPCR and Western Blotting (*n* = 3). **C** HT29 cells were treated with caspase 6 siRNA (Si1 and Si2) and Z-veid-fmk individually, followed by treatment with RIPK1/RIPK3/MLKL activator 1 for 24 h to assess the protein expression of p-MLKL and p-RIPK3. **D** Following treatment with caspase 6 siRNA or inhibitors, the cells were incubated with RIPK1/RIPK3/MLKL Activator 1 for 24 h, and the cellular viability was assessed using the CCK8 assay (*n* = 4). **E** QPCR and Western Blotting were employed to measure the expression levels of RIPK1 in HT29 cells following knockdown and treatment with inhibitors (*n* = 4). **F** The expression of RIPK1 was detected through immunoprecipitation with an anti-caspase 6 antibody. **G**–**J** IHC staining and quantitative analysis were employed to detect the expression level of RIPK1 in the colon of *Casp6* KO/Wt and Flox/*Casp6* cKO mice (*n* = 7). Scale bar = 50 μm. * *P* < 0.05, ** *P* < 0.01, and *** *P* < 0.001.

To investigate the cause of necroptosis in IECs after caspase 6 deficiency, we treated HT29 cells (a type of colorectal adenocarcinoma cell) with a specific necroptosis activator (RIPK1/RIPK3/MLKL activator 1). Our findings indicate that the treatment with 10 μM RIPK1/RIPK3/MLKL activator 1 resulted in approximately 50% HT29 cell death (Fig. 6A). Moreover, the combination of caspase 6 inhibitors (Z-veid-fmk, a specific inhibitor of caspase 6 activity) further enhanced HT29 cell mortality (Fig. 6A). Additionally, PI staining was employed to label deceased cells, and the findings further indicated that Z-veid-fmk augmented cell death induced by RIPK1/RIPK3/MLKL activator 1 (Fig. S5A, B). Conversely, siRNA was employed to suppress the expression of caspase 6 in HT29 cells (Fig. 6B), and subsequent examination revealed an elevation in the expression of p-MLKL and p-RIPK3 following caspase 6 knockdown (Fig. 6C). Furthermore, the combined application of caspase 6 siRNA and RIPK1/RIPK3/MLKL activator 1 was observed to augment the rate of cell death (Fig. 6D). These findings reaffirm that *Casp6* KO induces necroptosis, potentially mediated by the proteolytic activity of caspase 6.

Next, we explore how the knockdown of caspase 6 specifically promotes necroptosis. Our data suggest that caspase 6 is necessary for its enzymatic activity in regulating necroptosis. Previous studies have indicated that caspase 6 potentially regulates the intrinsic cleavage of RIPK1 [30]. Therefore, we investigated the impact of caspase 6 intervention on the expression of RIPK1. Our findings revealed that both the caspase 6 inhibitor and siRNA led to an elevation in the protein content of RIPK1 in the cellular remnants, while no notable alteration was observed at the RNA level (Fig. 6E), which aligns with the protein-level enzymatic cleavage function of caspase 6. Furthermore, the co-immunoprecipitation assay (Fig. 6F) revealed that caspase 6 did not directly bind to RIPK1, providing additional evidence that the impact of caspase 6 on RIPK1 was dependent on its enzyme activity. Lastly, we assessed the expression of RIPK1 in *Casp6* KO/Wt and Flox/*Casp6* cKO mice, revealing that both *Casp6* KO and cKO led to the over expressions of RIPK1 in colon cells (Fig. 6G–J). Therefore, we hypothesize that *Casp6* KO in IECs increases the protein levels of RIPK1, leading to the promotion of necroptosis.

### Knocking out caspase 6 impairs bacterial clearance by macrophages, and promotes bacterial translocation, consequently exacerbating IBDs

**Fig. 7:**
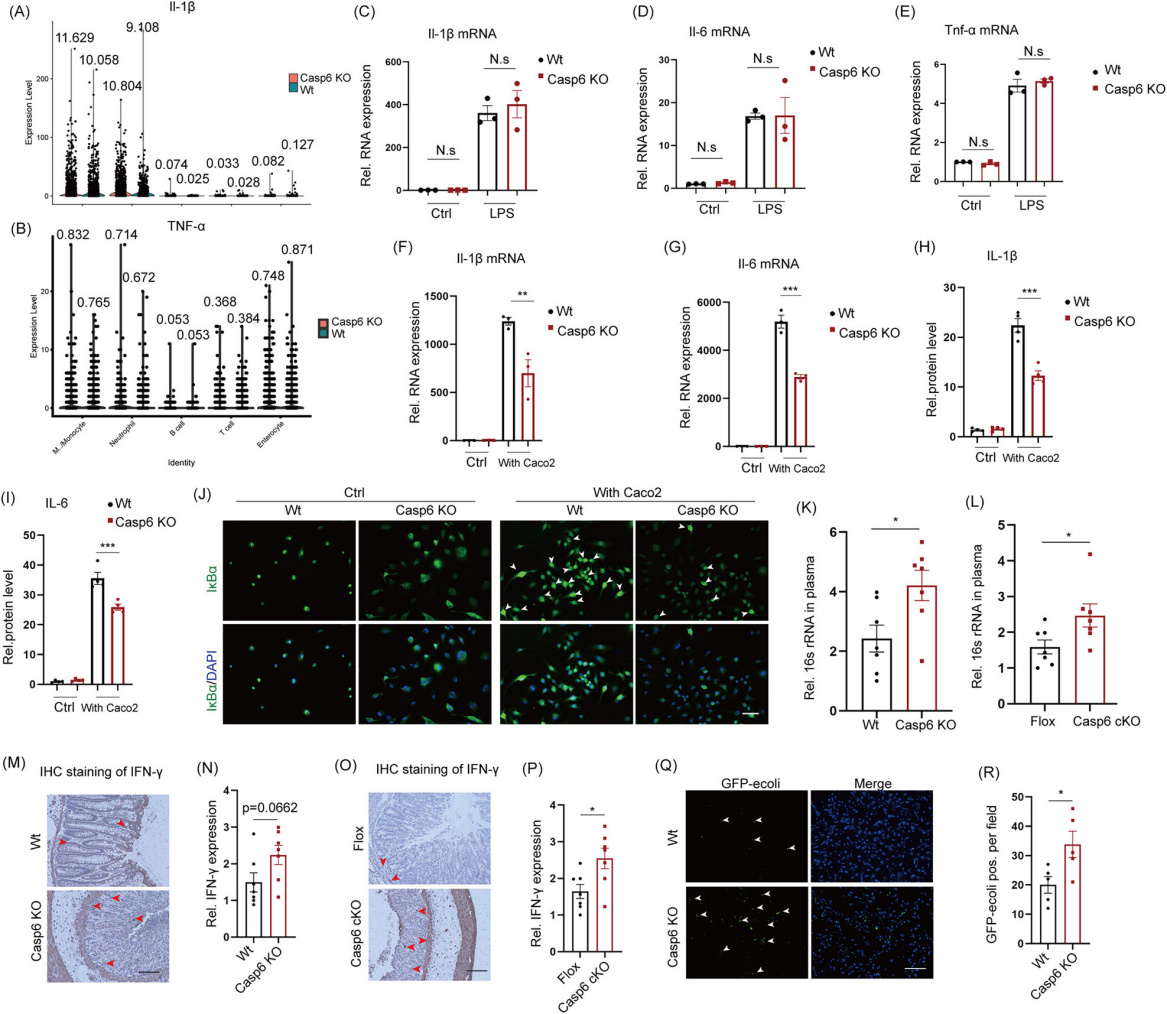
*Casp6* KO decreases macrophage clearance of bacteria, promotes bacterial translocation, and exacerbates IBD. **A**, **B** ScRNA-seq data demonstrates that *Casp6* KO affects the expression of the inflammatory cytokines Il-1β and Tnf-α. **C**-**E** LPS was employed to stimulate BMDM, and the mRNA expression levels of the inflammatory factors Il-1β, Il-6, and Tnf-α were quantified using q-PCR (*n* = 3). **F**, **G** Caco-2 cells were treated with H_2_O_2_ to simulate DAMPs. Subsequently, these cells were co-cultured with *Casp6* KO and Wt BMDM, and the expression of pro-inflammatory factors Il-1β and IL-6 in macrophages was assessed using q-PCR (*n* = 3). **H**, **I** The levels of IL-1β and IL-6 expression in the supernatant of BMDM were measured using enzyme-linked immunosorbent assay (*n* = 4). **J** The expression and subcellular localization of IκBα, a key protein in the NF-κB signaling pathway, were detected using immunofluorescence. Scale bar = 50 μm. **K**, **L** Plasma levels of 16 s rRNA were quantified using q-PCR (*n* = 7). **M**–**P** The level of INF-γ in colon tissue was assessed using IHC (*n* = 7). Scale bar = 50 μm. **Q**, **R** On the 7th day after the IBD model was established, *Escherichia coli* expressing GFP was administered orally into the intestine. Liver tissues were then sectioned and examined under a fluorescence microscope to detect bacterial colonization (*n* = 5). Scale bar = 50 μm. * *P* < 0.05, ** *P* < 0.01, and *** *P* < 0.001.

It is widely recognized that macrophage activation plays a crucial role in the development of IBDs. However, the regulatory function of caspase 6 in macrophage activation in the context of IBDs has yet to be elucidated. Analysis of the scRNA-seq data revealed a significant increase in the expression levels of Il-1β and Tnf-α following *Casp6* KO (Fig. 7A, B), indicating a positive correlation with disease severity. To investigate the factors contributing to the upregulation of Il-1β and Tnf-α following *Casp6* KO, we subjected bone marrow-derived macrophages (BMDMs) from *Casp6* KO and Wt mice to LPS stimulation. Interestingly, we observed no significant difference in LPS-induced expression of pro-inflammatory factors between *Casp6* KO and Wt mice (Fig. 7C–E). In addition, we investigated whether caspase 6 functions as a potential regulator of macrophage activation in response to damage-associated molecular patterns (DAMPs). Caco-2 cells were treated with H_2_O_2_ to simulate DAMPs-induced injury and were subsequently co-cultured with *Casp6* KO and Wt BMDMs. The result showed that *Casp6* KO macrophages significantly reduced the expression of Il-1β and Il-6 mRNA in Caco-2 cells (Fig. 7F, G). Previous studies have demonstrated that caspase 6 activates pan-apoptosis[19], leading to the cleavage of pro-inflammatory factors and increased protein expression of Il-1β. We assessed the levels of IL-1β and IL-6 in the supernatants of BMDMs and observed a reduction in their secretion in the presence of *Casp6* KO (Fig. 7H, I). In order to investigate the underlying cause of the reduced expression of pro-inflammatory factor RNA caused by *Casp6* KO after co-cultivation with Caco-2, we employed immunofluorescence to examine the expression and subcellular distribution of NF-кB signaling. Our findings demonstrate that *Casp6* KO markedly decreased the expression of IκBα protein induced by Caco2, suggesting that NF-κB might have been activated and translocated to the nucleus (Fig. 7J). These results suggest that *Casp6* KO diminishes the expression of pro-inflammatory factor RNA and protein induced by DAMPs. Furthermore, the exacerbation of IBDs by *Casp6* KO indicates that caspase 6 is not an intrinsic factor in macrophage inflammatory response.

Previous research has demonstrated that a deficiency of caspase 6 can impair the bactericidal capacity of macrophages [31]. Additionally, bacterial components such as flagellin and peptidoglycan have been found to interact with pattern recognition receptors on host cells, notably Toll-like receptors (TLRs). This interaction has the potential to induce an inflammatory response in the gut, thus contributing to the pathogenesis of IBDs in mice [32]. Consequently, compromising the bactericidal capacity of macrophages may exacerbate the development of IBDs. For this purpose, we initially examined the expression of plasma 16 s rRNA in the *Casp6* KO and Wt groups, as well as in Flox and *Casp6* cKO mice. The results demonstrated a significant increase in the expression level of plasma 16 s rRNA in both *Casp6* KO and cKO groups (Fig. 7K, L). IFN-γ can enhance the permeability of the intestinal epithelium, and the resulting impairment of the epithelial barrier function can exacerbate intestinal diseases by allowing excessive antigens and microorganisms to enter the mucosa [33]. Additionally, our findings revealed a significant elevation in the IFN-γ level in colon tissues of both *Casp6* KO and cKO groups (Fig. 7M–P). Ultimately, to effectively respond to bacterial translocation, we employed a modified intestinal loop experiment [34]. In this experiment, *Escherichia coli* expressing GFP was introduced into the intestine following a 7-day period of IBD model establishment, after which liver tissue was collected and prepared as frozen sections to assess bacterial colonization. The results demonstrated that *Casp6* KO resulted in a significant increase in bacterial colonization in the liver (Fig. 7Q, R). These findings indicate that *Casp6* KO leads to a weakened ability of macrophages to clear bacteria, thereby increasing bacterial translocation. This is also a contributing factor for the exacerbation of IBDs.

### *Casp 6* KO reduces bactericidal activity in a cathepsin L (CTSL)-dependent manner

**Fig. 8:**
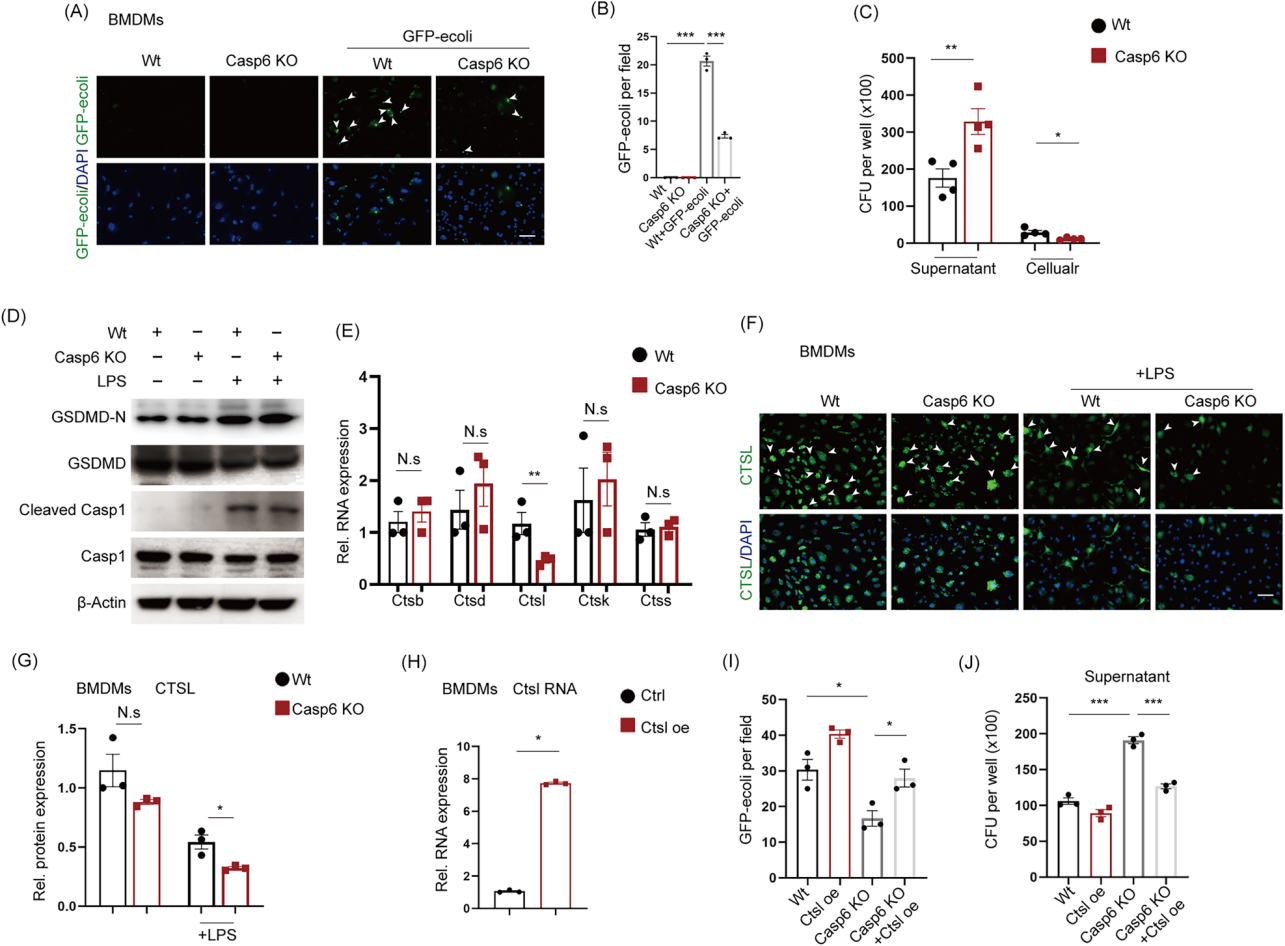
*Casp 6* KO reduces bactericidal activity in a CTSL-dependent manner. **A**, **B** Infect GFP-e.coli BMDMs and evaluate the phagocytic function (*n* = 3). Scale bar = 50 μm. **C** Analysis of bacterial counts in the supernatant and within the cells (*n* = 4). **D** WB analysis of the expression levels of proteins related to the pyroptosis pathway. **E** QPCR analysis of the RNA expression levels of lysosomal cathepsins Ctsb, Ctsd, Ctsl, Ctsk, and Ctss (*n* = 3). **F**, **G** Representative immunofluorescence image and quantitative analysis of CTSL protein (green) (*n* = 3). Scale bar = 50 μm. **H**–**J** Overexpress Ctsl, then reinfect with GFP-*E. coli*, and assess the phagocytic clearance of bacteria by BMDMs (*n* = 3). *P < 0.05, **P < 0.01, and ***P < 0.001.

To investigate the mechanism by which caspase 6 affects bacterial clearance, we co-cultured GFP-*E. coli* with BMDMs and measured the residual bacterial load after 24 h. Under the same multiplicity of infection, *Casp6* KO significantly impaired the phagocytic ability of macrophages toward GFP-*E.* *coli* compared to the Wt group (Fig. 8A, B), resulting in an approximately twofold increase in residual bacteria in the culture medium (Figs. S6A and 8C). This indicates that *Casp6* KO markedly interferes with the clearance of typical enteric bacteria by BMDMs. What is the underlying mechanism of this phenomenon? The caspase family may modulate the antibacterial defense of immune cells through multiple pathways, such as caspase 1-mediated pyroptosis [35], apoptosis [36], which can affect the functional state of macrophages, and the expression of lysosomal cathepsins, which may crosstalk with the caspase 1 pyroptosis pathway to influence macrophage function [37]. Our analysis first confirmed that knockout of caspase 6 does not affect bacteria-induced apoptosis. Furthermore, Western blotting for pyroptosis-related marker proteins showed that *Casp6* KO had minimal impact on the expression of GSDMD-N and cleaved caspase 1 (Fig. 8D). Subsequently, qPCR analysis revealed that in the *Casp6* KO group, the expression of Ctsl—a key enzyme involved in pathogen degradation—was significantly reduced (Fig. 8E). Immunofluorescence analysis also demonstrated that, under LPS stimulation, CTSL protein expression was decreased in the Casp6 KO group (Fig. 8F, G). To further clarify whether CTSL is the critical factor underlying the reduced bactericidal activity of Casp6 KO macrophages, we overexpressed CTSL in macrophages using adenovirus-mediated gene transduction (GeneChem, China), followed by GFP-*E. coli* infection. Overexpression of Ctsl successfully restored the bactericidal activity (Fig. 8H–J). These findings suggest that *Casp6* KO impairs bactericidal capacity in a CTSL-dependent manner. Nonetheless, the specific molecular mechanisms underlying this phenomenon require further investigation.

## Discussion

Caspase 6 is categorized as an effector or executioner caspase, capable of mediating both linear immunity and activation of the inflammasome [27, 38]. Nevertheless, the role of caspase 6 in IBDs remains uncertain. Our findings revealed a positive correlation between caspase 6 expression and the severity of disease progression in patients with clinical IBDs. Based on our initial hypothesis, we anticipated that a DSS-induced mouse model of IBDs, following *Casp6* KO, would ameliorate the inflammatory condition associated with IBDs. However, contrary to our initial expectation, we discovered that the absence of caspase 6 exacerbated the IBD condition, leading to increased necrosis of IECs and aggravated intestinal injury. Our research results indicate that caspase 6 effectively inhibits the necroptosis pathway of RIPK/MLKL by modulating the intrinsic cleavage function of RIPK1. When caspase 6 activity is inhibited, RIPK1 and RIPK3 activities are stabilized, and the downstream MLKL is recruited, leading to uncontrolled necroptosis, increased death of IECs, and amplified intestinal inflammation in IBDs. Our findings have important implications for preventing and treating the progression of IBDs.

The dysfunction of the epithelial barrier caused by the loss of caspase 6 activity is a significant factor in the etiology and pathology of IBDs. Decreased barrier function leads to increased risk of infection and microbial colonization [5, 39]. Thus, it is imperative to investigate the heterogeneity of IECs in patients with IBDs and identify the crucial mechanisms for enhancing IBD prognosis. In this study, we conducted scRNA-seq on the colons of *Casp6* KO mice to analyze and characterize the gene expression profiles of each cell type. Importantly, scRNA-seq revealed that *Casp6* KO resulted in damage to IECs, including necroptosis taking place in enterocytes, a reduced number of enteroendocrine cells, and stem cell damage. The cell types in the gut primarily consist of absorptive enterocytes, hormone-producing enteroendocrine cells, mucus-secreting goblet cells, antimicrobial and stem cell niche–providing Paneth cells, and cluster cells that are less well-defined, among others [40]. Enterocytes, which constitute the majority of cells in the epithelial layer, play a crucial role in absorbing nutrients and safeguarding the body against the hostile bacteria-rich environment [41, 42]. Necroptosis of enterocytes induced by mouse *Casp6* KO can disrupt the balance of cell death. Consequently, this condition elevates intestinal permeability and impairs barrier function, thereby contributing to the development of a broad range of acute and chronic intestinal diseases, including IBDs, colorectal cancer, and intestinal ischemia-reperfusion injury [43]. The elevated levels of RIPK1, RIPK3, and MLKL in the affected tissues of patients diagnosed with Crohn’s and ulcerative colitis a potential association between necroptosis and an elevated risk of developing IBDs [44]. Thus, it is plausible that the inhibition of caspase 6 activity is implicated in the underlying mechanism contributing to the heightened risk of developing IBDs. Additionally, the knockout of caspase 6 in mice resulted in a decrease in the quantity of intestinal endocrine cells. Enteroendocrine cells, accounting for about 1% of the entire intestinal epithelium, are the key sensors of intestinal microbiota and microbial metabolites, and play an important role in mucosal immunity, intestinal barrier function, visceral hyperalgesia, and gastrointestinal motility. Numerous studies have demonstrated an increasing recognition of the potential significance of interactions between enteroendocrine cells and gut microbiota in the development of IBDs [28, 44, 45]. The disruption of enteroendocrine cells by caspase 6 will undoubtedly impact the role of these cells in intestinal diseases. Finally, scRNA-seq results also revealed that caspase 6 deficiency leads to damage in intestinal stem cells. Due to constant exposure to the contents of the intestine, the intestinal epithelium necessitates rapid renewal to uphold the integrity of the mucosal barrier. This process relies on the proliferation and differentiation of intestinal stem cells [46]. IBDs are characterized by persistent inflammation and damage to the mucosal lining. Continuous infiltration of inflammatory cells and exposure to cytokines cause the death of intestinal stem cells and hinder epithelial regeneration [47, 48]. It can be inferred that in the context of IBDs, inhibiting caspase 6 activity can exacerbate intestinal inflammation by damaging intestinal stem cells. Based on the aforementioned findings, maintaining caspase 6 activity in IBDs can help prevent additional dysregulation of epithelial homeostasis.

Deficiency of caspase 6 exacerbates IBDs by upregulating the protein expression of RIPK1, thereby promoting necroptosis. Necroptosis is initiated through the sequential activation of RIPK1 and RIPK3, followed by the subsequent phosphorylation of MLKL, leading to the activation of downstream effectors [49, 50]. RIPK1 plays a pivotal role in the regulation of cell death by modulating caspase 8-mediated apoptosis or facilitating RIPK3-MLKL-dependent necroptosis in the absence of apoptosis [51, 52]. Blocking RIPK1 activity completely inhibited necroptosis [53, 54]. It is noteworthy that caspase 6 expression in cells can facilitate apoptosis by cleaving RIPK1, thus inhibiting the production of inflammatory factors and preventing necroptosis [19]. While caspase 8 and caspase 10 are capable of cleaving RIPK1 at higher concentrations, their impact is significantly weaker compared to caspase 6’s cleavage ability of RIPK1 [30]. In our study, through scRNA-seq differential gene expression analysis, we observed a significant increase in the expression of key genes involved in necroptosis, namely RIPK1, RIPK2, and MLKL, in the *Casp6* KO group. At the cellular level, we provided evidence that *Casp6* KO in IECs leads to the activation of the necroptosis pathway, which is attributed to the loss of RIPK1-targeted cleavage. Consistent with the findings of van Raam et al. [30]. Our results reaffirmed the involvement of caspase 6 in the regulation of RIPK1. This effect is crucial in preventing the progression of IBDs. Furthermore, Claudia et al. found that deletion of epithelial caspase 8 also induced the same necroptosis event [4]. Additionally, caspase 8 is involved in the cleavage of a subset of RIPKs, and its inhibition facilitates necroptosis [55]. Nevertheless, studies have demonstrated that inhibiting caspase 6 activity leads to a decrease in caspase 8 activation [56]. Therefore, we hypothesize that this mechanism could contribute to the enhancement of *Casp6* KO-induced necroptosis in IECs.

The activity of caspase 6 in macrophages did not contribute to the increased expression of inflammatory molecules caused by *Casp6* KO. However, the knockout of caspase 6 can impair the clearance of bacteria by macrophages, exacerbate bacterial translocation, and worsen IBDs. In our in vitro study, the LPS stimulation of BMDMs from *Casp6* KO mice did not alter the expression of proinflammatory cytokines induced by LPS when compared to the control group. This aligns with the research by Zheng et al., which showed that caspase 6 is not necessary for the activation of classical NLRP3, AIM2, NLRC4, and PYRIN inflammasomes, as well as caspase-11-mediated activation of non-classical NLRP3 inflammasomes under conditions of TLR2 priming and LPS transfection. Furthermore, a decrease in the formation of RIPK3 and ZBP1 complexes was observed in *Casp6* KO BMDMs, providing evidence for the interaction between caspase 6 and RIPK3 in promoting RIPK3’s interaction with ZBP1 [27]. ZBP1 is an important innate immune receptor for endogenous nucleic acid ligands and viral RNA products, and formation of the ZBP1-NLRP3 inflammasome is a key step in pyroptosis during IAV infection. ZBP1-NLRP3 inflammasome assembly requires caspase 6, and when caspase 6 is absent, NLRP3 activation is compromised following IAV infection. In cells lacking caspase 6, the cleavage of gasdermin D and caspase 1 is reduced, and the secretion of IL-18 and IL-1β is diminished [19]. This may explain the inflammation induced by DAMPs caused by the *Casp6* KO in our in vitro cell experiments. Similarly, in a study conducted by Hiroshi et al. [57], knockdown of caspase 6 only inhibited IκBα degradation and Tnf-α production in macrophages after co-culture with polymorphonucleous neutrophils. It did not affect the response of macrophages to LPS stimulation. In other words, the pathway regulated by caspase 6 in macrophages operates independently of TLR signaling. However, our scRNA-seq data demonstrated a significant increase in the expression levels of IL-1β and TNF-α following *Casp6* KO. The activation of RIPK1 and RIPK3 directly regulates pro-inflammatory signaling [58]. Together with our findings, this indicates that in vivo activation of RIPKs kinase-dependent necroptosis pathways in the IECs of *Casp6* knockout mice promotes the expression of inflammatory cytokines. Additionally, the extensive necrosis of IECs in vivo allows pathogens to breach the epithelial barrier and invade the intestinal mucosa, which can trigger a recognition response by macrophages equipped with TLRs, pattern recognition receptors, and other components. This recognition response further induces a cascade of pathway reactions, including the activation of the NLRP3 inflammasome, thus amplifying the inflammatory effect [59]. Our study found that *Casp6* KO and cKO mice exhibited significantly elevated expression of 16 s rRNA in plasma and IFN-γ in the colon, along with increased bacterial colonization in the liver. Similar to the investigation conducted by Alexander et al., caspase 6-deficient mice exhibited significantly elevated expression levels of IFN-γ and proinflammatory factors, such as IL-12 and Tnf-α, compared to their WT counterparts. These disparities were attributed to the secondary impact of varying bacterial loads, due to the heightened vulnerability to bacterial infections resulting from caspase 6 deletion [31].

Our findings reveal that *Casp6* KO significantly impairs macrophage bactericidal activity through a CTSL-dependent mechanism. CTSL is a crucial lysosomal cysteine protease that mediates the degradation of phagocytosed microbes and regulates antigen processing, thereby influencing both innate and adaptive immune responses [60]. The decreased bactericidal activity observed in *Casp6* KO macrophages may be attributable to disrupted CTSL expression, maturation, or enzymatic activity, resulting in inefficient phagolysosomal function and impaired pathogen clearance. This is consistent with previous evidence highlighting the essential role of CTSL in controlling pathogen infections and participate in the innate immune response [61, 62]. Furthermore, based on our research results, it can be inferred that caspase 6 may modulate CTSL activity either directly, through proteolytic processing, or indirectly, by regulating the transcription of lysosomal proteases or the trafficking of lysosomal vesicles. This caspase 6-CTSL axis appears to be critical for maintaining intestinal immune homeostasis, as its disruption leads to persistent microbial burden and exacerbates mucosal inflammation, characteristic of IBD. These results not only clarify a novel mechanism linking cell death pathways and lysosomal proteolysis in host defense but also highlight the therapeutic potential of targeting CTSL or its regulation by caspase 6 to enhance bacterial clearance and ameliorate intestinal inflammation in IBD patients.

## Conclusions

In conclusion, our findings indicate that caspase 6-mediated cleavage of RIPK1 plays a vital role in controlling the cell death pathway and deciding the fate of IECs in IBDs. Notably, we further demonstrate that *Casp6* KO not only diminishes macrophage capability to eliminate bacteria, but does so in a CTSL-dependent manner, leading to greater intestinal bacterial translocation. These results reveal a critical caspase 6–CTSL axis in the regulation of mucosal immunity and bacterial clearance. Thus, our findings have significant implications for the clinical management of IBDs. While caspase 6 activation in IBDs typically facilitates IECs apoptosis and accelerates cell shedding, indiscriminate anti-apoptosis treatment may trigger IECs necroptosis and exacerbate IBDs. Considering the potential benefits of modestly modulating caspase 6 activity to maintain both appropriate epithelial turnover and effective antibacterial responses, future therapies should aim to regulate apoptosis, prevent necroptosis, and preserve the integrity of the caspase 6–CTSL-mediated antibacterial defense.

## Materials and methods

### Patients and samples

Intestinal tissues were obtained from the inflamed regions of 10 IBD patients through surgical procedures. Inclusion criteria were: patients aged 18–65 years, diagnosed with active IBD based on clinical manifestations, endoscopic, and pathological findings, and meeting the diagnostic standards for IBD established by the Inflammatory Bowel Disease Group of the Chinese Society of Gastroenterology in 2023. Exclusion criteria included the use of conventional IBD medications (such as immunosuppressants, salicylates, corticosteroids, or biologics) within the past three months, the presence of other autoimmune diseases, abnormal routine stool test results, or a history of intestinal surgery. The control group consisted of individuals without evidence of IBD, as confirmed by clinical evaluation and endoscopic examination, and was matched to the IBD group according to relevant criteria. The study protocol was approved by the Ethics Committee of the Second Xiangya Hospital of Central South University (2021JJ40844), and informed consent was obtained from all participants.

### Animal model

Heterozygous caspase 6-deficient (caspase 6^+/−^) mice and heterozygous IEC-specific *Casp6* KO (Flox/caspase 6^+/−^) mice on C57BL/6 J background obtained from Cyagen (Guangzhou, China) were used to generate homozygous targeted mice by crossbreeding the heterozygous targeted mice and expanded breeding. All animals were maintained under specific pathogen-free conditions at a temperature of 23 ± 2 °C and a light/dark cycle of 12 h each. All animals were provided with ad libitum access to both drinking water and food, with 4 mice housed per cage. The mice used in the experiments were between 6 and 8 weeks of age.

Mouse model of IBDs. Mice with both genes and Wt mice were divided into 3 groups of 6 mice each according to genotype. Autoclaved water was prepared by adding DSS (MW 36,000–50,000 Da, Lot No.) at a concentration of 2.5%. The mice were allowed to drink this water freely for 7 days. The water was changed every 2 days during the 7 days. After that, it was replaced with autoclaved water without DSS, and the mice were given 2 days to drink freely before being anesthetized and euthanized. The disease activity index (DAI) was calculated based on the scoring system, as shown in Supplementary Table S7.

### Cell culture

The HT29 cell line (SUNNCELL, China), Caco-2 cell line (SUNNCELL, China), and BMDMs were cultured in Dulbecco’s Modified Eagle Medium (DMEM; Gibco, USA) supplemented with 10% fetal bovine serum (Biological Industries, Israel) and 1% penicillin-streptomycin solution (Biological Industries, Israel). The culture medium was changed according to the specific growth requirements of each cell type. All cells were incubated at 37 °C in a humidified atmosphere with 5% CO₂. For macrophage cultures, M-CSF (ABclonal, China) was added at a final concentration of 20 ng/ml.

Extraction of primary BMDMs: 6- to 10-week-old mice were sacrificed by cervical dislocation, and both femurs and tibias were harvested. All muscle tissue surrounding the bones was carefully removed, retaining only the bone shafts. Using a 5 ml syringe filled with sterile PBS, the bone marrow cavities were flushed with a needle, and the bone marrow cells were collected into centrifuge tubes. This procedure was repeated several times until the inside of the bones appeared white. The resulting cell suspension was filtered through a 70 µm cell strainer to remove bone debris. The cells were centrifuged at 1000 rpm for 5 min, after which the supernatant was discarded with a pipette, leaving the red cell pellet. Subsequently, 10 ml of D-PBS was added slowly to resuspend and wash the cells, followed by another centrifugation under the same conditions. The washing process was performed twice in total. Finally, bone marrow-derived mononuclear cells were collected and cultured in complete medium containing 20 ng/ml M-CSF.

### Small intestine H&E score

After 9 days, the entire colon was surgically removed, and its length was measured. The colon was then cleaned, fixed with a 4% paraformaldehyde solution, and embedded in paraffin. Tissue sections were stained with H&E. Histologic scoring was performed by a pathologist in a blinded fashion. The scoring system is presented in Supplementary Table S8.

### Bacteria culture

The bacteria utilized in this study were Nissle1917-superfolder green fluorescent protein (sfGFP) (GFP-*E. coli*) (BioSci, China), a fluorescently labeled derivative of the probiotic *E. coli* Nissle 1917. This strain was engineered through CRISPR genome editing to stably integrate an sfGFP expression cassette into the *E. coli* Nissle 1917 chromosome, thereby enabling reliable fluorescent labeling for subsequent in vivo and in vitro tracking analyses. The strain was cultured in Luria-Bertani liquid medium at 37 °C with shaking at 200 rpm, and bacterial growth was monitored by measuring the optical density at 600 nm (OD600). The working concentration of GFP-*E. coli* was adjusted to 1 × 10^9^ CFU/mL.

For the bacterial phagocytosis assay in macrophages [63], BMDMs were seeded onto coverslips placed in 24-well plates. After a 12-h incubation period, GFP-labeled *E. coli* (2.5 × 10^7^ bacteria per well) were added to each well and incubated with the cells for 90 min. Subsequently, the cells were analyzed by confocal microscopy.

In the hepatic colonization assay, after IBD modeling, mice were administered 200 μL of GFP-*E. coli* by oral gavage. Twenty-four hours later, the mice were sacrificed, and liver tissues were collected under sterile conditions. Liver tissue sections were then examined using fluorescence microscopy to assess the presence and distribution of GFP-positive bacteria.

### QPCR

QPCR was performed using BrightCycle Universal SYBR Green qPCR Mix with UDG (RK21219, ABclonal, China) and LightCycler® 480 instrument (Roche Applied Science). The ratios were normalized with externally taught standards. Primers were designed with the following primer sequences: Il-1β (mouse) (forward, TGCCACCTTTTGACAGTGATG, reverse, AAGGTCCACGGGAAAGACAC), Tnf-α (mouse) (forward, AGGCACTCCCCCAAAAGATG, reverse, CCACTTGGTGGTTTGTGAGTG), caspase 6 (mouse) (forward, AAGTGTTCGATCCAGCCGAG, reverse, CAGGTTGTCTCTGTCTGCGT), Ripk1 (mouse) (forward, TCCTTAGAGGAGGACCAGCG, reverse, GGAGTTCGGTGCTGAAGTGG), Il-6 (mouse) (forward, CAACGATGATGCACTTGCAGA, reverse, TGTGACTCCAGCTTATCTCTTGG), 16 s rRNA (mouse) (forward, TCGTCGGCAGCGTCAGATGTGTATAAGAGACAGCCTACGGGNGGCWGCAG, reverse, GTCTCGTGGGCTCGGAGATGTGTATAAGAGACAGGACTACHVGGGTATCTAATCC), Tnf-α (human) (forward, GACAAGCCTGTAGCCCATGT, reverse, GGAGGTTGACCTTGGTCTGG), β-actin (mouse) (forward, CACTGTCGAGTCGCGTCC, reverse, TCATCCATGGCGAACTGGTG), β-actin (human) (forward, CCTCGCCTTTGCCGATCC, reverse, CCATCACGCCCTGGTGC). Ctsl (mouse) (forward, ACTCGGAGGAGTCTTACCCC, reverse, CTGGAGAGACGGATGGCTTG), Ctsb (mouse) (forward, GGCTCTTGTTGGGCATTTGG, reverse, ACTCGGCCATTGGTGTGAAT), Ctsd (mouse) (forward, AATCCCTCTGCGCAAGTTCA, reverse, AATCCCTCTGCGCAAGTTCA), Ctsk (mouse) (forward, CTCCAGTCAAGAACCAGGGC, reverse, CCGTTCTGCTGCACGTATTG), Ctss (mouse) (forward, CCACGCTGCCATCAGAAGAT, reverse, TTTTCCCAGATGAGACGCCG), The q-PCR amplification protocol was as follows: initial denaturation at 95 °C for 30 s, followed by 40 cycles of 95 °C for 5 s and 60 °C for 30 s. After amplification, a melting curve analysis was performed with the following steps: 95 °C for 5 s, 65 °C for 60 s, and 97 °C for 1 s. Finally, the reaction was cooled at 37 °C for 30 s.

### Transferase-mediated dUTP nick-end-labeling (TUNEL) assay

TUNEL staining was performed to detect apoptotic cells using a commercially available TUNEL assay kit (Beyotime, China), strictly adhering to the manufacturer’s protocol. In brief, paraffin-embedded tissue sections were first incubated at 60 °C for 1 h to ensure optimal adhesion and softening. Subsequently, the slides underwent deparaffinization in xylene, followed by a graded ethanol series to achieve rehydration. To permeabilize the tissue and facilitate the removal of proteins, sections were treated with proteinase K solution at 37 °C for 30 min. After thorough rinsing with phosphate-buffered saline (PBS) to eliminate residual enzyme, the TUNEL reaction mixture was applied evenly to each section. The samples were incubated in a humidified chamber at 37 °C for 1 h to allow for effective labeling of DNA strand breaks. Following incubation, the slides were washed twice with PBS to remove unbound reagents. Nuclear counterstaining and mounting were accomplished using an antifade mounting medium containing DAPI (Invitrogen, USA) to visualize cell nuclei. Finally, fluorescence images were acquired and documented utilizing a Zeiss fluorescence microscope, enabling the assessment and quantification of apoptotic cells within the tissue samples.

### ScRNA-seq

As mentioned in the previous article [64]. First, the harvested mouse small intestine tissues were carefully cut into approximately 1 mm³ pieces. The tissue fragments were then subjected to enzymatic digestion using the MACS Tumor Dissociation Kit Mouse (Miltenyi Biotec, Germany) at 37 °C for 30 min to ensure thorough dissociation into single cells. Following digestion, the resulting cell suspension was sequentially filtered through 70 and 40 μm cell strainers (BD, USA) to remove residual tissue debris and obtain a high-quality single-cell suspension. The filtered cells were collected by centrifugation at 300 × *g* for 10 min, after which the supernatant was discarded.

To eliminate erythrocyte contamination, the cell pellet was resuspended in erythrocyte lysis buffer (Thermo Fisher Scientific, USA) and incubated at room temperature for 2 min. The reaction was promptly terminated, and cells were centrifuged again to remove lysed red blood cells. The resulting cell pellet was washed twice with PBS containing 0.04% BSA to further purify the cell population and minimize debris.

Subsequently, cDNA amplification and library preparation were performed according to the protocols provided with the MGI DNBelab C Series reagent kit (MGI, China). The single-cell suspensions were encapsulated into droplets, facilitating the separation and barcoding of individual cells. After droplet emulsions were broken, mRNA-capturing magnetic beads were collected. Reverse transcription was then carried out to synthesize cDNA from captured mRNA, followed by amplification and purification steps to ensure the generation of high-quality cDNA libraries.

The quality and quantity of the resulting libraries were assessed using the Qubit ssDNA Assay Kit (Thermo Fisher Scientific, USA) and the Agilent Bioanalyzer 2100. Only libraries meeting the required standards were selected for further processing. High-throughput sequencing was subsequently performed on the DIPSEQ T1 platform. The raw sequencing data were filtered and processed using the DNBelab C Series scRNA analysis software to generate gene expression matrices, which served as the foundation for downstream single-cell transcriptomic analyses.

### FITC-dextran permeability assay

To assess intestinal permeability in vivo, fluorescein isothiocyanate (FITC)-dextran (4 kDa; Sigma–Aldrich, USA) was utilized as a tracer. FITC-dextran was freshly dissolved in sterile PBS at a concentration corresponding to a dose of 100 mg/kg body weight. The prepared solution was administered to the experimental mice via oral gavage according to the experimental design. After 1.5 h post-gavage, the animals were anesthetized and sacrificed. Blood samples were promptly collected by cardiac puncture into EDTA-coated tubes. The collected blood was then centrifuged at 3000 rpm for 10 min at 4 °C to separate the plasma.

For quantitative analysis, 100 µl of plasma from each animal was transferred into individual wells of a black 96-well microplate. The fluorescence intensity was measured using a microplate spectrofluorometer (Thermo Fisher Scientific, USA) with excitation/emission wavelengths set at 485 nm and 520 nm, respectively. The plasma fluorescence values obtained from mice that did not receive FITC-dextran served as controls and were subtracted from experimental readings to eliminate background signals. The degree of intestinal permeability was expressed as the relative percentage of FITC-dextran fluorescence in plasma, normalized to the negative control group.

### Western blot analysis

Total protein was extracted from tissue or cell samples using RIPA lysis buffer containing protease and phosphatase inhibitors, and protein concentrations were determined with a BCA protein assay kit (Thermo Fisher Scientific, USA). Equal amounts of protein (typically 20–40 µg per well) were mixed with loading buffer and denatured by boiling at 95 °C for 5 min. According to the molecular weight of the target proteins, samples were separated by SDS-polyacrylamide gel electrophoresis (SDS-PAGE) on 4–20% polyacrylamide gradient gels (ACE, China). After electrophoresis, proteins were transferred onto polyvinylidene difluoride membranes (Millipore, USA) using a wet transfer system (Bio-Rad Mini Trans-Blot, USA) at 4 °C and 100 V for 90 min. The membranes were then blocked with 5% (w/v) skim milk in Tris-buffered saline containing 0.1% Tween-20 (TBST) at room temperature for 90 min to prevent nonspecific binding. After blocking, membranes were incubated overnight at 4 °C with primary antibodies diluted in TBST containing 5% bovine serum albumin (BSA) or skim milk, following the manufacturer’s instructions. The next day, membranes were washed three times with TBST and incubated with species-appropriate horseradish peroxidase-conjugated secondary antibodies at room temperature for 45 min. After thorough washing with TBST, protein bands were visualized using enhanced chemiluminescence reagents (Thermo Fisher Scientific, USA). Detailed information about the antibodies can be found in Supplementary Table S6.

### Immunohistochemistry (IHC) and immunofluorescence

For IHC, 4 μm paraffin sections were deparaffinized and subjected to antigen retrieval in sodium citrate buffer (10 mM sodium citrate and 0.05% Tween 20, pH 6.0) at 96 °C for 20 min. The sections were then processed using an immunohistochemistry kit (Abcam, UK), incubated with peroxidase for 10 min, washed twice with PBS, treated with a protein blocking solution, and washed again. Slides were placed in a humidified chamber, incubated with the primary antibody at 4 °C overnight. The following day, after removal of the primary antibody, the appropriate secondary antibody was applied. The tissues were then washed and incubated with DAB chromogenic solution. Upon completion of color development, the slides were rinsed under running water for 10 min. Nuclei were counterstained with hematoxylin for 10 min, and excess stain was removed under running water. After dehydration with absolute ethanol and xylene, the sections were mounted with neutral resin and observed under a microscope.

For immunofluorescence, the procedures were essentially the same as for immunohistochemistry. After antigen retrieval, the sections were blocked with 5% goat serum, followed by incubation with the primary antibody overnight at 4 °C. On the next day, Alexa Fluor 488 or 594-conjugated secondary antibodies were added and incubated at room temperature for 60 min. The sections were then washed three times with PBS, incubated with DAPI containing an anti-fade agent (Invitrogen, USA), and observed under a microscope. Details of the antibodies are provided in Supplementary Table S6.

### Small interfering RNA (siRNA)

Cells were seeded in 6-well plates at approximately 50% confluency for transfection, gently swirled in a figure-8 pattern, and incubated overnight. On the following day, cell status was assessed under UV-sterilized conditions prior to transfection. Two experimental groups were prepared (control and siRNA) using four 1.5 mL microcentrifuge tubes. For each group, 10 µL siRNA-NC (control) or siRNA was mixed with 140 µL Opti-MEM I to generate Solution A, while Solution B consisted of 7 µL Lipofectamine™ RNAiMAX combined with 143 µL Opti-MEM I. Solutions A and B were combined and incubated for 10 min at room temperature. During this period, the cell culture medium was replaced with antibiotic-free medium following PBS washes. The resulting complexes were then added to the cells. After gentle swirling, the cells were incubated for 36 h prior to harvesting for Western blot, RNA-seq, or qRT-PCR, or subsequent drug treatment. Throughout the procedure, sterility was maintained, and siRNA was kept on ice. The RNAi targeting sequences of the indicated molecules: caspase 6-Si1 (CGAUUGCUUCAUCUGUGUCUUTT), caspase 6-Si2 (AAGACACAGAUGAAGCAAUCGTT).

### Statistical analysis

All values are reported as mean ± standard error. Statistical analysis was carried out using GraphPad Prism 8 software. Differences between the two groups were assessed using an unpaired Student’s *t*-test under the assumption of normality and equal variances; if these conditions were not met, a nonparametric alternative was used. For comparisons involving more than two groups, one-way ANOVA was applied to evaluate statistical significance. Differences in means between groups were considered statistically significant at *P* < 0.05.

## Supplementary information

Supplementary figures and legends

Supplementary table legends

Supplementary Table S1

Supplementary Table S2

Supplementary Table S3

Supplementary Table S4

Supplementary Table S5

Supplementary Table S6

Supplementary Table S7

Supplementary Table S8

Original western blot

## Data Availability

The datasets generated during and/or analysed during the current study are available from the corresponding author upon reasonable request.

## References

[1] Zhang Y, Li X, Luo Z, Ma L, Zhu S, Wang Z, et al. ECM1 is an essential factor for the determination of M1 macrophage polarization in IBD in response to LPS stimulation. Proc Natl Acad Sci USA. 2020;117:3083–92. 31980528 10.1073/pnas.1912774117 PMC7022174

[2] Agrawal M, Jess T. Implications of the changing epidemiology of inflammatory bowel disease in a changing world. U Eur Gastroenterol J. 2022;10:1113–20. 10.1002/ueg2.12317 PMC9752308 36251359

[3] Xavier RJ, Podolsky DK. Unravelling the pathogenesis of inflammatory bowel disease. Nature. 2007;448:427–34. 17653185 10.1038/nature06005

[4] Günther C, Buchen B, He GW, Hornef M, Torow N, Neumann H, et al. Caspase-8 controls the gut response to microbial challenges by Tnf-α-dependent and independent pathways. Gut. 2015;64:601–10. 25379949 10.1136/gutjnl-2014-307226 PMC4392221

[5] Peterson LW, Artis D. Intestinal epithelial cells: regulators of barrier function and immune homeostasis. Nat Rev Immunol. 2014;14:141–53. 24566914 10.1038/nri3608

[6] Jozawa H, Inoue-Yamauchi A, Arimura S, Yamanashi Y. Loss of C/EBPδ enhances apoptosis of intestinal epithelial cells and exacerbates experimental colitis in mice. Genes Cells Devoted Mol Cell Mechan. 2019;24:619–26. 10.1111/gtc.12711 31233664

[7] Di Sabatino A, Ciccocioppo R, Luinetti O, Ricevuti L, Morera R, Cifone MG, et al. Increased enterocyte apoptosis in inflamed areas of Crohn’s disease. Dis Colon Rectum. 2003;46:1498–507. 14605569 10.1007/s10350-004-6802-z

[8] Zeissig S, Bojarski C, Buergel N, Mankertz J, Zeitz M, Fromm M, et al. Downregulation of epithelial apoptosis and barrier repair in active Crohn’s disease by tumour necrosis factor alpha antibody treatment. Gut. 2004;53:1295–302. 15306588 10.1136/gut.2003.036632 PMC1774168

[9] Zhang J, Cen L, Zhang X, Tang C, Chen Y, Zhang Y, et al. MPST deficiency promotes intestinal epithelial cell apoptosis and aggravates inflammatory bowel disease via AKT. Redox Biol. 2022;56:102469. 36126419 10.1016/j.redox.2022.102469 PMC9486620

[10] Wang R, Li H, Wu J, Cai ZY, Li B, Ni H, et al. Gut stem cell necroptosis by genome instability triggers bowel inflammation. Nature. 2020;580:386–90. 32296174 10.1038/s41586-020-2127-x

[11] Patankar JV, Müller TM, Kantham S, Acera MG, Mascia F, Scheibe K, et al. E-type prostanoid receptor 4 drives resolution of intestinal inflammation by blocking epithelial necroptosis. Nat Cell Biol. 2021;23:796–807. 34239062 10.1038/s41556-021-00708-8

[12] Zhou M, He J, Shi Y, Liu X, Luo S, Cheng C, et al. ABIN3 negatively regulates necroptosis-induced intestinal inflammation through recruiting A20 and restricting the ubiquitination of RIPK3 in inflammatory bowel disease. J Crohn’s Colitis. 2021;15:99–114. 32599618 10.1093/ecco-jcc/jjaa131

[13] Roberts JZ, Crawford N, Longley DB. The role of ubiquitination in apoptosis and necroptosis. Cell Death Differ. 2022;29:272–84. 34912054 10.1038/s41418-021-00922-9 PMC8817035

[14] Kerr JF, Wyllie AH, Currie AR. Apoptosis: a basic biological phenomenon with wide-ranging implications in tissue kinetics. Br J Cancer. 1972;26:239–57. 4561027 10.1038/bjc.1972.33 PMC2008650

[15] McIlwain DR, Berger T, Mak TW. Caspase functions in cell death and disease. Cold Spring Harb Perspect Biol. 2013;5:a008656. 23545416 10.1101/cshperspect.a008656 PMC3683896

[16] Ruemmele FM, Seidman EG, Lentze MJ. Regulation of intestinal epithelial cell apoptosis and the pathogenesis of inflammatory bowel disorders. J Pediatr Gastroenterol Nutr. 2002;34:254–60. 11964947 10.1097/00005176-200203000-00005

[17] Williams JM, Duckworth CA, Watson AJ, Frey MR, Miguel JC, Burkitt MD, et al. A mouse model of pathological small intestinal epithelial cell apoptosis and shedding induced by systemic administration of lipopolysaccharide. Dis Models Mechan. 2013;6:1388–99. 10.1242/dmm.013284 PMC3820262 24046352

[18] Newton K. RIPK1 and RIPK3: critical regulators of inflammation and cell death. Trends Cell Biol. 2015;25:347–53. 25662614 10.1016/j.tcb.2015.01.001

[19] Qi L, Wang L, Jin M, Jiang M, Li L, Li Y. Caspase-6 is a key regulator of cross-talk signal way in PANoptosis in cancer. Immunology. 2023;169:245–59. 36814103 10.1111/imm.13633

[20] Welz PS, Wullaert A, Vlantis K, Kondylis V, Fernández-Majada V, Ermolaeva M, et al. FADD prevents RIP3-mediated epithelial cell necrosis and chronic intestinal inflammation. Nature. 2011;477:330–4. 21804564 10.1038/nature10273

[21] Pierdomenico M, Negroni A, Stronati L, Vitali R, Prete E, Bertin J, et al. Necroptosis is active in children with inflammatory bowel disease and contributes to heighten intestinal inflammation. Am J Gastroenterol. 2014;109:279–87. 24322838 10.1038/ajg.2013.403

[22] Becker C, Watson AJ, Neurath MF. Complex roles of caspases in the pathogenesis of inflammatory bowel disease. Gastroenterology. 2013;144:283–93. 23219999 10.1053/j.gastro.2012.11.035

[23] Holler N, Zaru R, Micheau O, Thome M, Attinger A, Valitutti S, et al. Fas triggers an alternative, caspase-8-independent cell death pathway using the kinase RIP as effector molecule. Nat Immunol. 2000;1:489–95. 11101870 10.1038/82732

[24] Vercammen D, Beyaert R, Denecker G, Goossens V, Van Loo G, Declercq W, et al. Inhibition of caspases increases the sensitivity of L929 cells to necrosis mediated by tumor necrosis factor. J Exp Med. 1998;187:1477–85. 9565639 10.1084/jem.187.9.1477 PMC2212268

[25] Salvesen GS, Walsh CM. Functions of caspase 8: the identified and the mysterious. Semin Immunol. 2014;26:246–52. 24856110 10.1016/j.smim.2014.03.005 PMC4099255

[26] Dupaul-Chicoine J, Yeretssian G, Doiron K, Bergstrom KS, McIntire CR, LeBlanc PM, et al. Control of intestinal homeostasis, colitis, and colitis-associated colorectal cancer by the inflammatory caspases. Immunity. 2010;32:367–78. 20226691 10.1016/j.immuni.2010.02.012

[27] Zheng M, Karki R, Vogel P, Kanneganti TD. Caspase-6 is a key regulator of innate immunity, inflammasome activation, and host defense. Cell. 2020;181:674–87.e13. 32298652 10.1016/j.cell.2020.03.040 PMC7425208

[28] Atanga R, Singh V, In JG. Intestinal enteroendocrine cells: present and future druggable targets. Int J Mol Sci. 2023;24:8836. 10.3390/ijms24108836 PMC10218851 37240181

[29] Chen Y, Ye Z, Seidler U, Tian D, Xiao F. Microenvironmental regulation of intestinal stem cells in the inflamed intestine. Life Sci. 2021;273:119298. 33667519 10.1016/j.lfs.2021.119298

[30] van Raam BJ, Ehrnhoefer DE, Hayden MR, Salvesen GS. Intrinsic cleavage of receptor-interacting protein kinase-1 by caspase-6. Cell Death Differ. 2013;20:86–96. 22858542 10.1038/cdd.2012.98 PMC3524638

[31] Bartel A, Göhler A, Hopf V, Breitbach K. Caspase-6 mediates resistance against Burkholderia pseudomallei infection and influences the expression of detrimental cytokines. PLoS ONE. 2017;12:e0180203. 28686630 10.1371/journal.pone.0180203 PMC5501493

[32] Serino M, Blasco-Baque V, Nicolas S, Burcelin R. Managing the manager: gut microbes, stem cells and metabolism. Diab Metab. 2014;40:186–90. 10.1016/j.diabet.2013.12.004 24462190

[33] Beaurepaire C, Smyth D, McKay DM. Interferon-gamma regulation of intestinal epithelial permeability. J Interferon Cytokine Res J Int Soc Interferon Cytokine Res. 2009;29:133–44. 10.1089/jir.2008.0057 19196071

[34] Sorribas M, Jakob MO, Yilmaz B, Li H, Stutz D, Noser Y, et al. FXR modulates the gut-vascular barrier by regulating the entry sites for bacterial translocation in experimental cirrhosis. J Hepatol. 2019;71:1126–40. 31295531 10.1016/j.jhep.2019.06.017

[35] Kayagaki N, Stowe IB, Lee BL, O’Rourke K, Anderson K, Warming S, et al. Caspase-11 cleaves gasdermin D for non-canonical inflammasome signalling. Nature. 2015;526:666–71. 26375259 10.1038/nature15541

[36] Hersh D, Monack DM, Smith MR, Ghori N, Falkow S, Zychlinsky A. The Salmonella invasin SipB induces macrophage apoptosis by binding to caspase-1. Proc Natl Acad Sci USA. 1999;96:2396–401. 10051653 10.1073/pnas.96.5.2396 PMC26795

[37] Arezki Y, Rapp M, Lebeau L, Ronzani C, Pons F. Cationic carbon nanoparticles induce inflammasome-dependent pyroptosis in macrophages via lysosomal dysfunction. Front Toxicol. 2022;4:925399. 35928766 10.3389/ftox.2022.925399 PMC9345407

[38] Mehl JL, Earle A, Lammerding J, Mhlanga M, Vogel V, Jain N. Blockage of lamin-A/C loss diminishes the pro-inflammatory macrophage response. iScience. 2022;25:105528. 36465100 10.1016/j.isci.2022.105528 PMC9708799

[39] Mankertz J, Schulzke JD. Altered permeability in inflammatory bowel disease: pathophysiology and clinical implications. Curr Opin Gastroenterol. 2007;23:379–83. 17545772 10.1097/MOG.0b013e32816aa392

[40] Gribble FM, Reimann F. Enteroendocrine cells: chemosensors in the intestinal epithelium. Annu Rev Physiol. 2016;78:277–99. 26442437 10.1146/annurev-physiol-021115-105439

[41] Gerbe F, Legraverend C, Jay P. The intestinal epithelium tuft cells: specification and function. Cell Mol Life Sci CMLS. 2012;69:2907–17. 22527717 10.1007/s00018-012-0984-7 PMC3417095

[42] Moor AE, Harnik Y, Ben-Moshe S, Massasa EE, Rozenberg M, Eilam R, et al. Spatial reconstruction of single enterocytes uncovers broad zonation along the intestinal villus axis. Cell. 2018;175:1156–67.e15. 30270040 10.1016/j.cell.2018.08.063

[43] Subramanian S, Geng H, Tan XD. Cell death of intestinal epithelial cells in intestinal diseases. Sheng li xue bao. Acta Physiol. Sin. 2020;72:308–24. PMC7755516 32572429

[44] Zhou P, Zhang S, Wang M, Zhou J. The induction mechanism of ferroptosis, necroptosis, and pyroptosis in inflammatory bowel disease, colorectal cancer, and intestinal injury. Biomolecules. 2023;13:820. 10.3390/biom13050820 PMC10216135 37238692

[45] Yu Y, Yang W, Li Y, Cong Y. Enteroendocrine cells: sensing gut microbiota and regulating inflammatory bowel diseases. Inflamm bowel Dis. 2020;26:11–20. 31560044 10.1093/ibd/izz217 PMC7539793

[46] Barker N. Adult intestinal stem cells: critical drivers of epithelial homeostasis and regeneration. Nat Rev Mol Cell Biol. 2014;15:19–33. 24326621 10.1038/nrm3721

[47] Ma L, Yu J, Zhang H, Zhao B, Zhang J, Yang D, et al. Effects of immune cells on intestinal stem cells: prospects for therapeutic targets. Stem Cell Rev Rep. 2022;18:2296–314. 35279803 10.1007/s12015-022-10347-7

[48] Hou Q, Huang J, Ayansola H, Masatoshi H, Zhang B. Intestinal stem cells and immune cell relationships: potential therapeutic targets for inflammatory bowel diseases. Front Immunol. 2020;11:623691. 33584726 10.3389/fimmu.2020.623691 PMC7874163

[49] Jorgensen I, Rayamajhi M, Miao EA. Programmed cell death as a defence against infection. Nat Rev Immunol. 2017;17:151–64. 28138137 10.1038/nri.2016.147 PMC5328506

[50] Remijsen Q, Goossens V, Grootjans S, Van den Haute C, Vanlangenakker N, Dondelinger Y, et al. Depletion of RIPK3 or MLKL blocks TNF-driven necroptosis and switches towards a delayed RIPK1 kinase-dependent apoptosis. Cell Death Dis. 2014;5:e1004. 24434512 10.1038/cddis.2013.531 PMC4040672

[51] Ofengeim D, Yuan J. Regulation of RIP1 kinase signalling at the crossroads of inflammation and cell death. Nat Rev Mol Cell Biol. 2013;14:727–36. 24129419 10.1038/nrm3683

[52] Rickard JA, O’Donnell JA, Evans JM, Lalaoui N, Poh AR, Rogers T, et al. RIPK1 regulates RIPK3-MLKL-driven systemic inflammation and emergency hematopoiesis. Cell. 2014;157:1175–88. 24813849 10.1016/j.cell.2014.04.019

[53] Degterev A, Hitomi J, Germscheid M, Ch’en IL, Korkina O, Teng X, et al. Identification of RIP1 kinase as a specific cellular target of necrostatins. Nat Chem Biol. 2008;4:313–21. 18408713 10.1038/nchembio.83 PMC5434866

[54] Sahoo G, Samal D, Khandayataray P, Murthy MK. A review on caspases: key regulators of biological activities and apoptosis. Mol Neurobiol. 2023;60:5805–37. 37349620 10.1007/s12035-023-03433-5

[55] Han J, Zhong CQ, Zhang DW. Programmed necrosis: backup to and competitor with apoptosis in the immune system. Nat Immunol. 2011;12:1143–9. 22089220 10.1038/ni.2159

[56] Rust C, Wild N, Bernt C, Vennegeerts T, Wimmer R, Beuers U. Bile acid-induced apoptosis in hepatocytes is caspase-6-dependent. J Biol Chem. 2009;284:2908–16. 19017654 10.1074/jbc.M804585200

[57] Kobayashi H, Nolan A, Naveed B, Hoshino Y, Segal LN, Fujita Y, et al. Neutrophils activate alveolar macrophages by producing caspase-6-mediated cleavage of IL-1 receptor-associated kinase-M. J Immunol. 2011;186:403–10. 21098228 10.4049/jimmunol.1001906 PMC3151149

[58] Najjar M, Saleh D, Zelic M, Nogusa S, Shah S, Tai A, et al. RIPK1 and RIPK3 kinases promote cell-death-independent inflammation by toll-like receptor 4. Immunity. 2016;45:46–59. 27396959 10.1016/j.immuni.2016.06.007 PMC4956514

[59] Sun S, Xu X, Liang L, Wang X, Bai X, Zhu L, et al. Lactic acid-producing probiotic Saccharomyces cerevisiae attenuates ulcerative colitis via suppressing macrophage pyroptosis and modulating gut microbiota. Front Immunol. 2021;12:777665. 34899735 10.3389/fimmu.2021.777665 PMC8652295

[60] Turk V, Stoka V, Vasiljeva O, Renko M, Sun T, Turk B, et al. Cysteine cathepsins: from structure, function and regulation to new frontiers. Biochim Biophys Acta. 2012;1824:68–88. 22024571 10.1016/j.bbapap.2011.10.002 PMC7105208

[61] Zhang N, Gao P, Yin B, Li J, Wu T, Kuang Y, et al. Cathepsin L promotes secretory IgA response by participating in antigen presentation pathways during Mycoplasma Hyopneumoniae infection. PLoS ONE. 2019;14:e0215408. 30986254 10.1371/journal.pone.0215408 PMC6464228

[62] Chen J, Zhang L, Yang N, Cao M, Tian M, Fu Q, et al. Characterization of the immune roles of cathepsin L in turbot (Scophthalmus maximus L.) mucosal immunity. Fish Shellfish Immunol. 2020;97:322–35. 31805413 10.1016/j.fsi.2019.12.005

[63] Liu Y, Huang Y, Yang W, Hu W, Wu Z, Wu T, et al. Aspartame enhances the scavenging activity of mice to low-dose Escherichia coli infection via strengthening macrophage phagocytosis caused by sweet taste receptor activation. FASEB J Publ Federation Am Soc Exp Biol. 2024;38:e70170. 10.1096/fj.202401396RR 39535424

[64] Wei ZX, Jiang SH, Qi XY, Cheng YM, Liu Q, Hou XY, et al. scRNA-seq of the intestine reveals the key role of mast cells in early gut dysfunction associated with acute pancreatitis. World J Gastroenterol. 2025;31:103094. 40182603 10.3748/wjg.v31.i12.103094 PMC11962851

