# Supplementary material for: Caspase 6 deficiency exacerbates inflammatory bowel disease via enterocyte necroptosis and bacterial translocation
Source: Cell Death Discov. 2025 Dec 13;12:59. doi: 10.1038/s41420-025-02877-z (PMC12848308; doi:10.1038/s41420-025-02877-z)
Supplement: Supplementary file 1 — Supplementary figures and legends [file 41420_2025_2877_MOESM1_ESM.pdf]

Supplementary figures

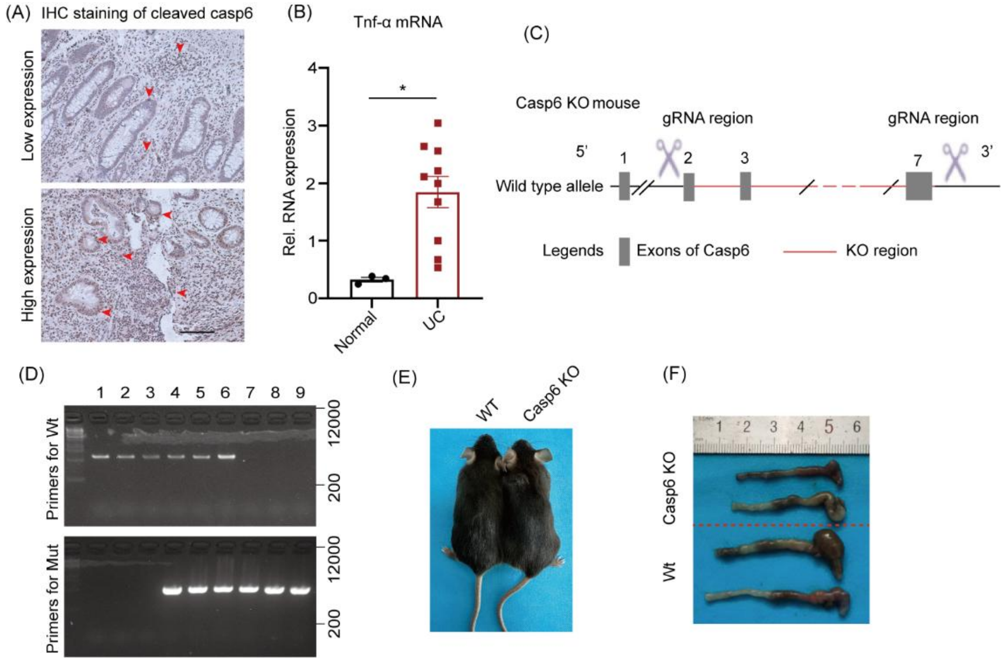

Figure S1. Detection of cleaved caspase 6 and Tnf-α expression levels, as well as the generation of caspase 6 knockout mice. A) Representative IHC staining images of cleaved caspase 6, showing high expression in the colonic tissues of patients with UC and low expression in those of healthy controls. Scale bar = 50 μm. B) The relative mRNA expression levels of Tnf-α were measured in colonic tissues from patients with ulcerative colitis and healthy controls (n=3-10). C-E) *Casp6* KO mice were generated. The strategy for *Casp6* KO, southern blot identification, and solid maps of Wt and *Casp6* KO mice (7-week-old) are presented. F) Colon length was compared between Wt and *Casp6* KO mice. * $P < 0.05$.

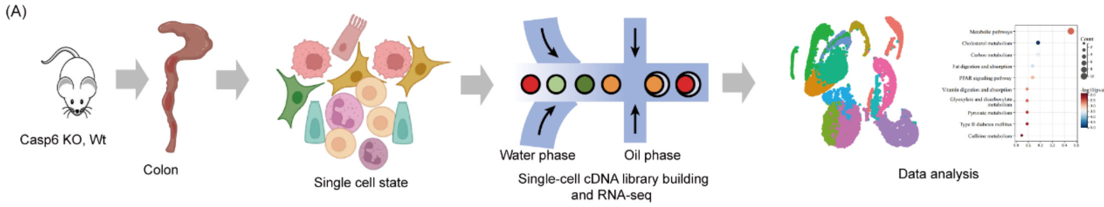

Figure S2. Workflow illustration of scRNA-seq analysis. A) Workflow illustration of scRNA-seq analysis in Wt and *Casp6* KO mouse colons.

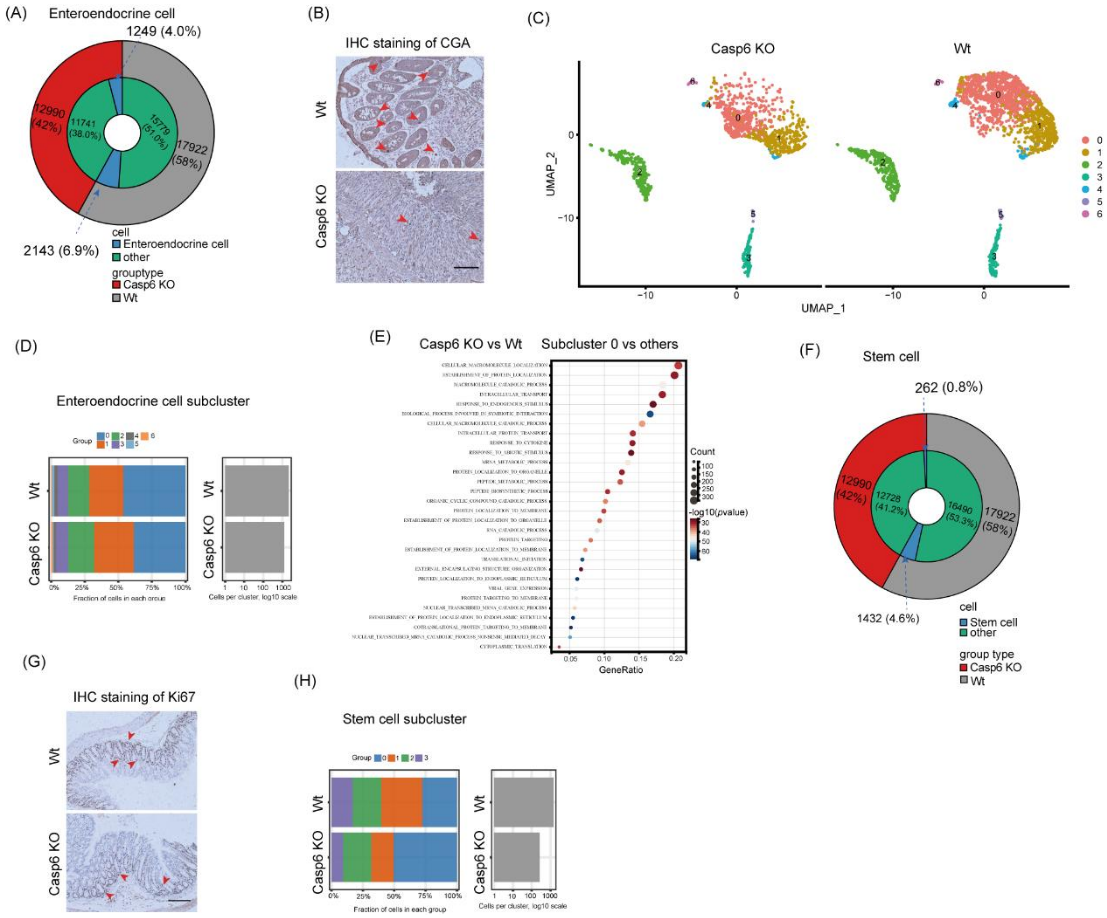

Figure S3. The effect of *Casp6* knockout on intestinal endocrine cells and stem cells. A) Alterations in the population of enteroendocrine cells between *Casp6* KO and Wt. B) IHC analysis was conducted to examine the expression of CGA in enteroendocrine cells. Scale bar = 50 μm. C) By employing the UMAP method for dimensionality reduction analysis, 7 distinct subpopulations of enteroendocrine cells were identified. D) The distribution of each subpopulation in *Casp6* KO and Wt enteroendocrine cells was determined. E) KEGG analysis was conducted to compare subcluster 0 with other subclusters. F) The alterations in the quantity of intestinal stem cells between *Casp6* KO and Wt mice were examined. G) IHC was employed to detect the expression of the cell proliferation marker Ki67 in the intestinal epithelium (n=7). Scale bar = 50 μm. H) The proportions of each subpopulation of intestinal stem cells in *Casp6* KO and Wt mice were determined.

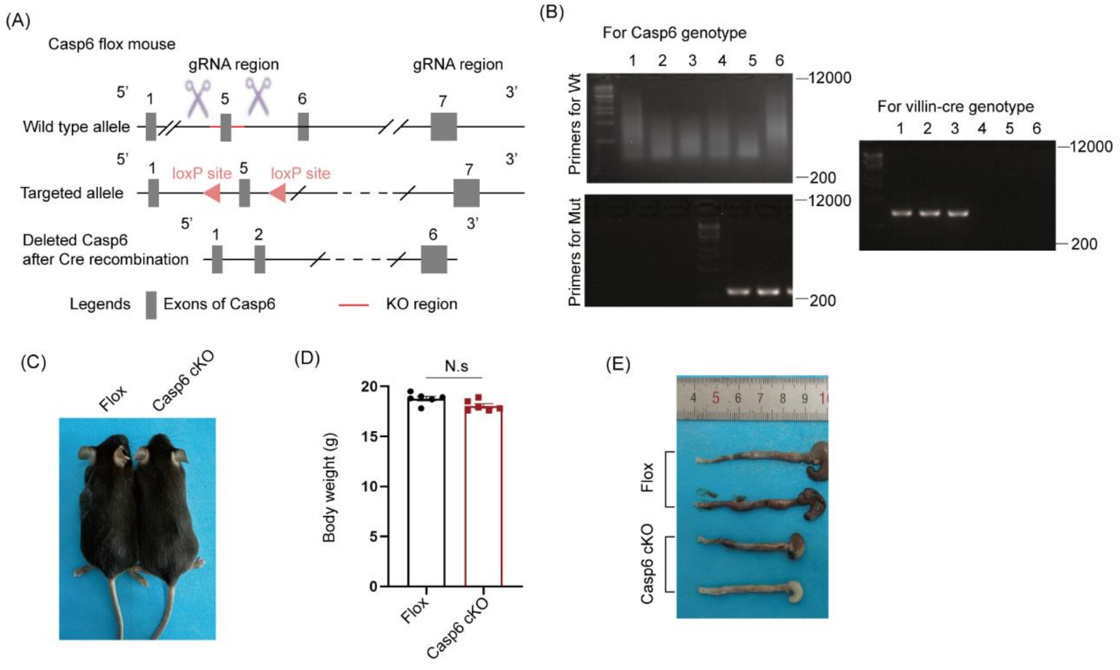

Figure S4. Generation and identification of Flox and cKO mice. A) Strategy for constructing *Casp6* Flox mice. B) Identifying cKO mice. C-D) Schematic diagram and weight measurements of 6-week-old mice (n=6). E) Colon length was compared between Flox and cKO mice.

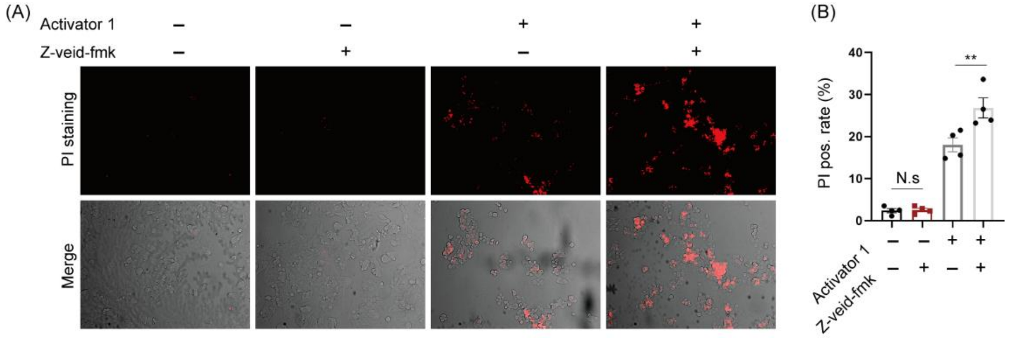

Figure S5. PI staining. A-B) Cells were stained with PI dye, and the number of PI-positive cells was quantified by capturing fluorescence microscope images (n=4). ** $P < 0.01$

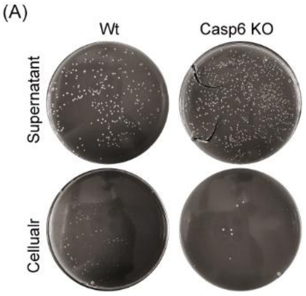

Figure S6. Bacterial enumeration. A) Analysis of bacterial counts in the supernatant and within the cells.
