# Supplementary material for: Caspase 6 deficiency exacerbates inflammatory bowel disease via enterocyte necroptosis and bacterial translocation
Source: Cell Death Discov. 2025 Dec 13;12:59. doi: 10.1038/s41420-025-02877-z (PMC12848308; doi:10.1038/s41420-025-02877-z)
Supplement: Supplementary file 2 — Supplementary table legends [file 41420_2025_2877_MOESM2_ESM.docx]

**Supplementary table legends**

**Table S1. Differentially expressed genes (DEGs)**

DEGs in enterocytes identified by scRNA sequencing. Readcout_1: Normalized read counts in the caspase 6 knockout group. Readcout_2: Normalized read counts in the wild-type group.

**Table S2. Markers of enterocytes**

The cell markers for each enterocyte subgroup were listed.

**Table S3. Gene Ontology (GO) enrichment analysis results for enteroendocrine cells.**

The differentiated expression genes of enteroendocrine between caspase 6 knockout and wild-type group were listed.

**Table S4. Markers of enteroendocrine**

The cell markers for each enteroendocrine cell subgroup were listed.

**Table S5. Markers of stem cells**

The cell markers for each intestinal stem cell subgroup were listed.

**Table S6. Antibodies used**

Details of the antibodies used.

**Table S7. Disease Activity Index (DAI)**

The DAI scoring system.

**Table S8. H&E score**

The HE staining scoring system.
