# Supplementary material for: Caspase 6 deficiency exacerbates inflammatory bowel disease via enterocyte necroptosis and bacterial translocation
Source: Cell Death Discov. 2025 Dec 13;12:59. doi: 10.1038/s41420-025-02877-z (PMC12848308; doi:10.1038/s41420-025-02877-z)
Supplement: Supplementary file 8 — Supplementary Table S6 [file 41420_2025_2877_MOESM8_ESM.docx]

**Supplementary Table S6**

**Details of antibodies used**

| **Antibodies** | **Manufacturer** | **location** | **Catalog No** | **Test** | **Concentration used** |
| --- | --- | --- | --- | --- | --- |
| Rabbit anti-cleaved caspase 6 | Immunoway | Plano, USA | YC0008 | IHC | 1:500/1:2000 |
| Rabbit anti-Phospho-RIPK3 | CST | Massachusetts, USA | 91702S | IF/WB | 1:1000 |
| Rabbit anti-Phospho-MLKL | Affinity | Jiangsu, China | AF7420 | IF/WB | 1:500/1:2000 |
| Rabbit anti-Chromogranin A | Proteintech | Illinois, USA | 10529-1-AP | IHC | 1:500/1:2000 |
| Rabbit anti-Ki67 | Abclonal | Wuhan, China | A2094 | IHC | 1:500/1:2000 |
| Rabbit anti-CD44 | Abclonal | Wuhan, China | A12410 | IF | 1:50/1:200 |
| Rabbit anti-caspase 6 | GeneTex | California, USA | GTX59553 | WB | 1:1000 |
| Mouse anti-RIPK3 | Santa | California, USA | sc-374639 | WB | 1:100/1:1000 |
| Rabbit anti-MLKL | Abclonal | Wuhan, China | A17312 | WB | 1:500/1:2000 |
| Rabbit anti-RIPK1 | Abclonal | Wuhan, China | A19580 | WB/IHC/IP | 1:500/1:1000 |
| Rabbit anti-IκBα | Abclonal | Wuhan, China | A19714 | IF | 1:500/1:2000 |
| Rabbit anti-IFN-γ | Abclonal | Wuhan, China | A12450 | IHC | 1:2000/1:10000 |
| Rabbit anti-β-actin | Proteintech | Illinois, USA | 81115-1-RR | WB | 1:5000/1:50000 |
| Mouse anti- caspase 1 | Santa | California, USA | sc-56036 | WB | 1:100/1:1000 |
| Rabbit anti-cleaved caspase 1 | CST | Massachusetts, USA | 89332S | WB | 1:1000 |
| Rabbit anti-GSDMD-N | FineTest | Wuhan, China | FNab10690 | WB | 1:200/1:2000 |
| Rabbit anti-GSDMD | Abcam | Cambridge, UK | ab219800 | WB | 1:1000/1:2000 |
| Rabbit anti-CTSL1 | Proteintech | Illinois, USA | 27952-1-AP | IF | 1:500 |
