# Supplementary material for: Caspase 6 deficiency exacerbates inflammatory bowel disease via enterocyte necroptosis and bacterial translocation
Source: Cell Death Discov. 2025 Dec 13;12:59. doi: 10.1038/s41420-025-02877-z (PMC12848308; doi:10.1038/s41420-025-02877-z)
Supplement: Supplementary file 9 — Supplementary Table S7 [file 41420_2025_2877_MOESM9_ESM.docx]

**Supplementary table S7**

**Scoring system for Disease Activity Index (DAI)**

| Score | Weight loss | Stool consistency | Blood stool |
| --- | --- | --- | --- |
| 0 | no loss | normal | no blood |
| 1 | 1-5% |  |  |
| 2 | 5-10% | Pasty stool | Occult blood positive |
| 3 | 10-20% |  |  |
| 4 | ＞20% | watery diarrhea | Obvious bloody stool |
