# Supplementary material for: Caspase 6 deficiency exacerbates inflammatory bowel disease via enterocyte necroptosis and bacterial translocation
Source: Cell Death Discov. 2025 Dec 13;12:59. doi: 10.1038/s41420-025-02877-z (PMC12848308; doi:10.1038/s41420-025-02877-z)
Supplement: Supplementary file 10 — Supplementary Table S8 [file 41420_2025_2877_MOESM10_ESM.docx]

**Supplementary table S8**

**H&E score**

| Score | inflammatory cell infiltration | Epithelial deletion | Crypt destruction | Goblet cells decrease |
| --- | --- | --- | --- | --- |
| 0 | no | no | no | no |
| 1 | mild | 1-5% | 1-5% | mild |
| 2 | moderate | 5-10% | 5-10% | moderate |
| 3 | severe | ＞10% | ＞10% | severe |
