# Supplementary material for: Caspase 6 deficiency exacerbates inflammatory bowel disease via enterocyte necroptosis and bacterial translocation
Source: Cell Death Discov. 2025 Dec 13;12:59. doi: 10.1038/s41420-025-02877-z (PMC12848308; doi:10.1038/s41420-025-02877-z)
Supplement: Supplementary file 11 — Original western blot [file 41420_2025_2877_MOESM11_ESM.pptx]

## Slide 1
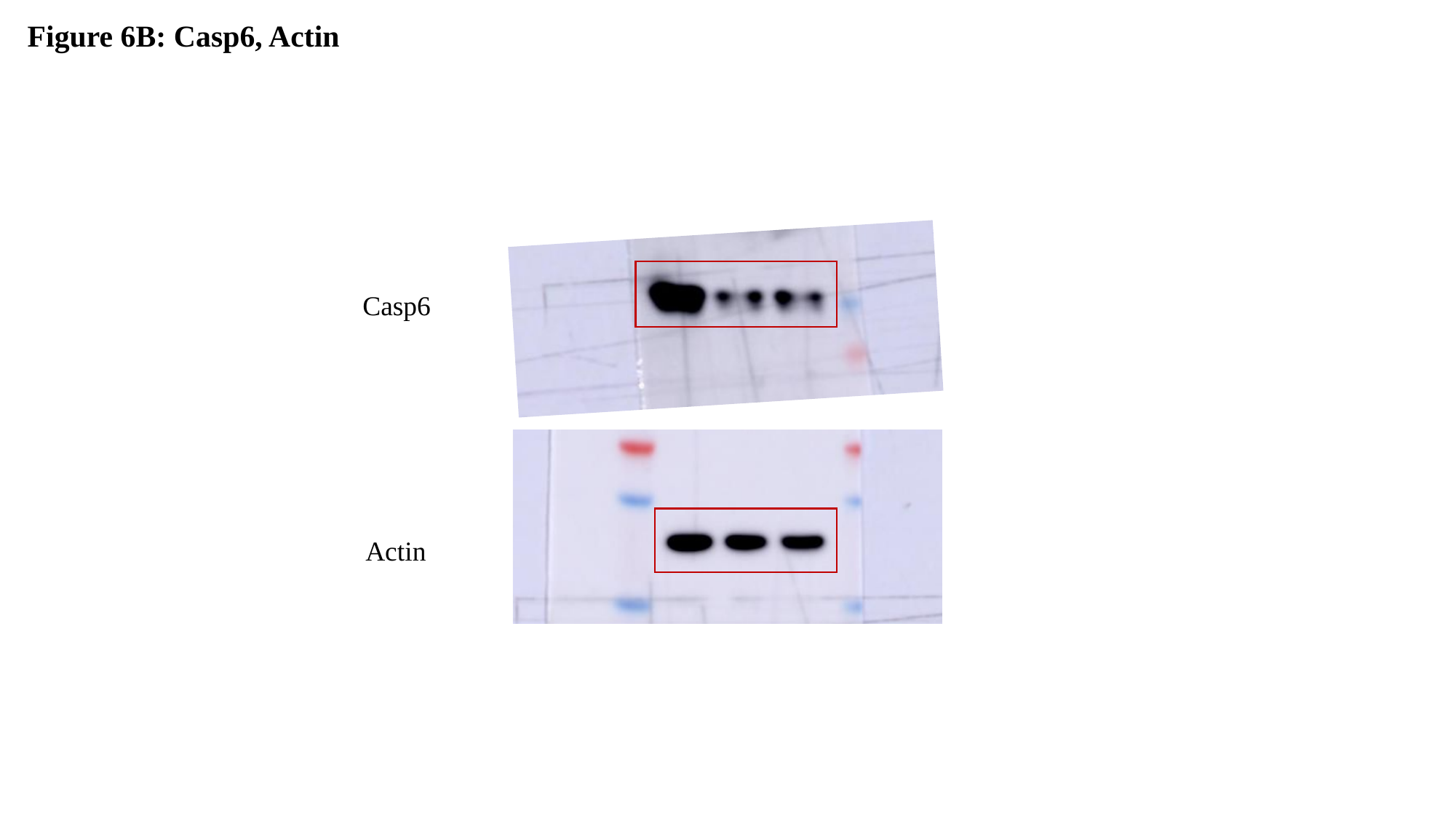

Figure 6B: Casp6, Actin
Casp6
Actin

## Slide 2
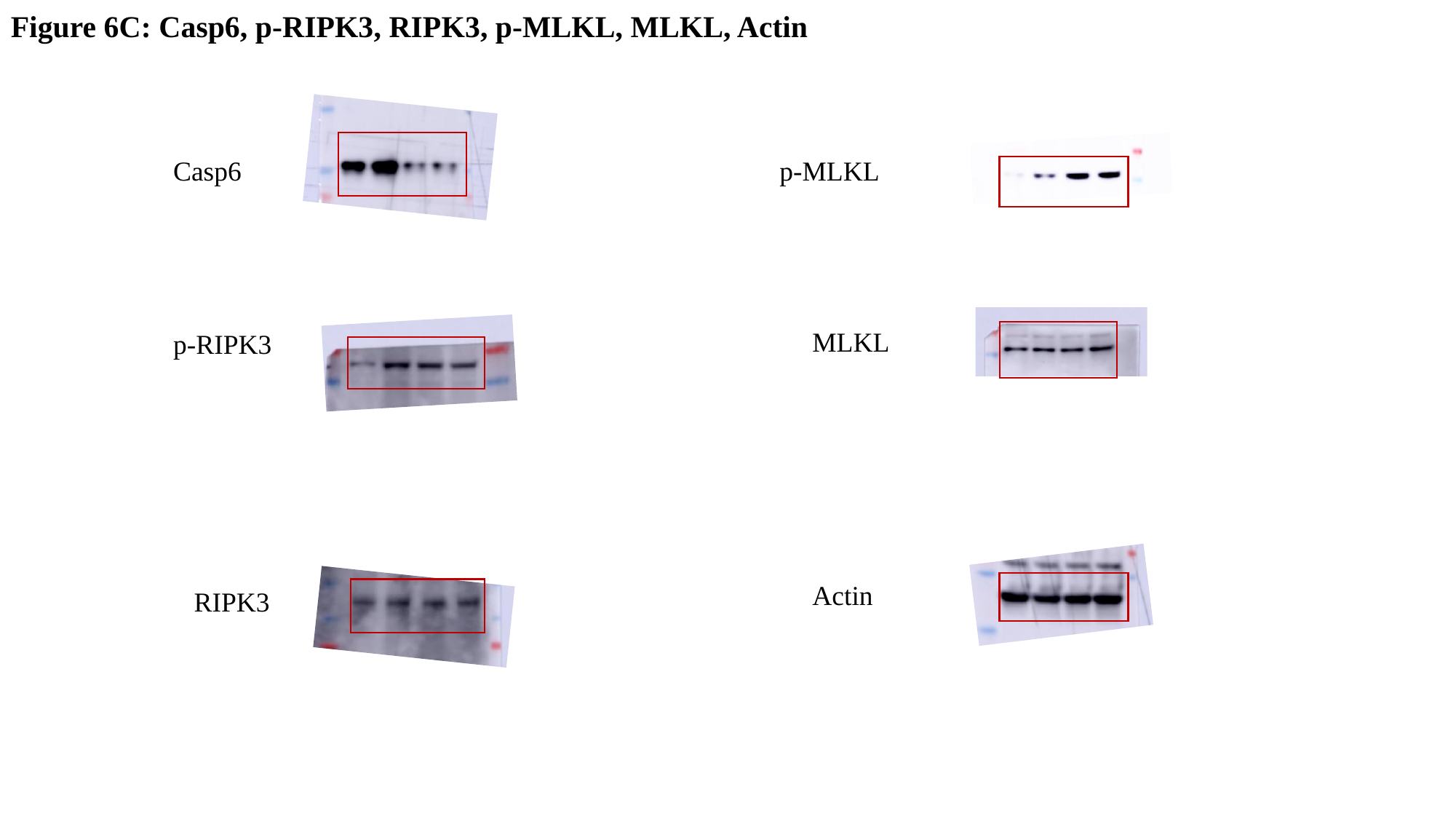

Figure 6C: Casp6, p-RIPK3, RIPK3, p-MLKL, MLKL, Actin
Casp6
p-MLKL
MLKL
p-RIPK3
Actin
RIPK3

## Slide 3
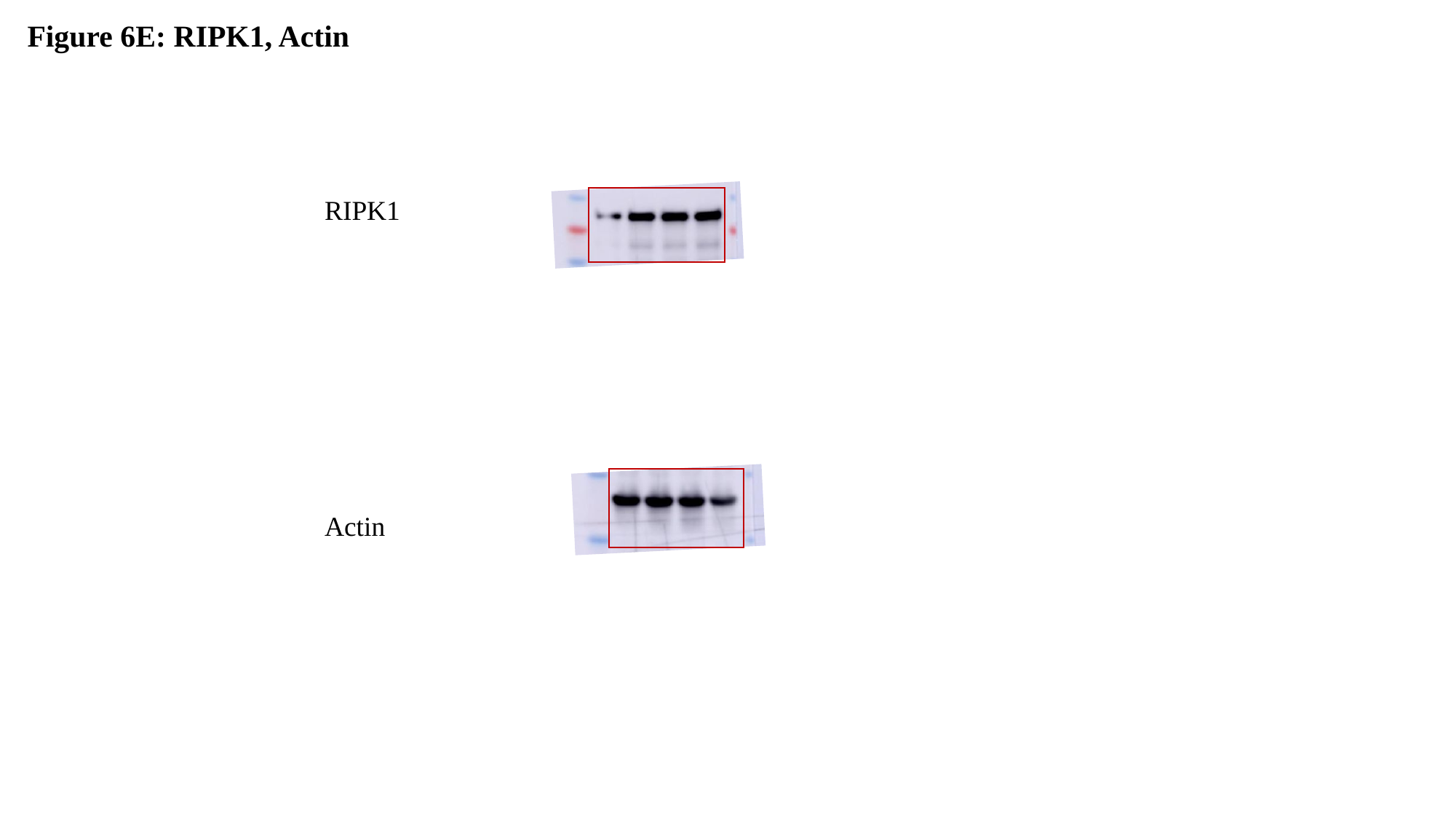

Figure 6E: RIPK1, Actin
RIPK1
Actin

## Slide 4
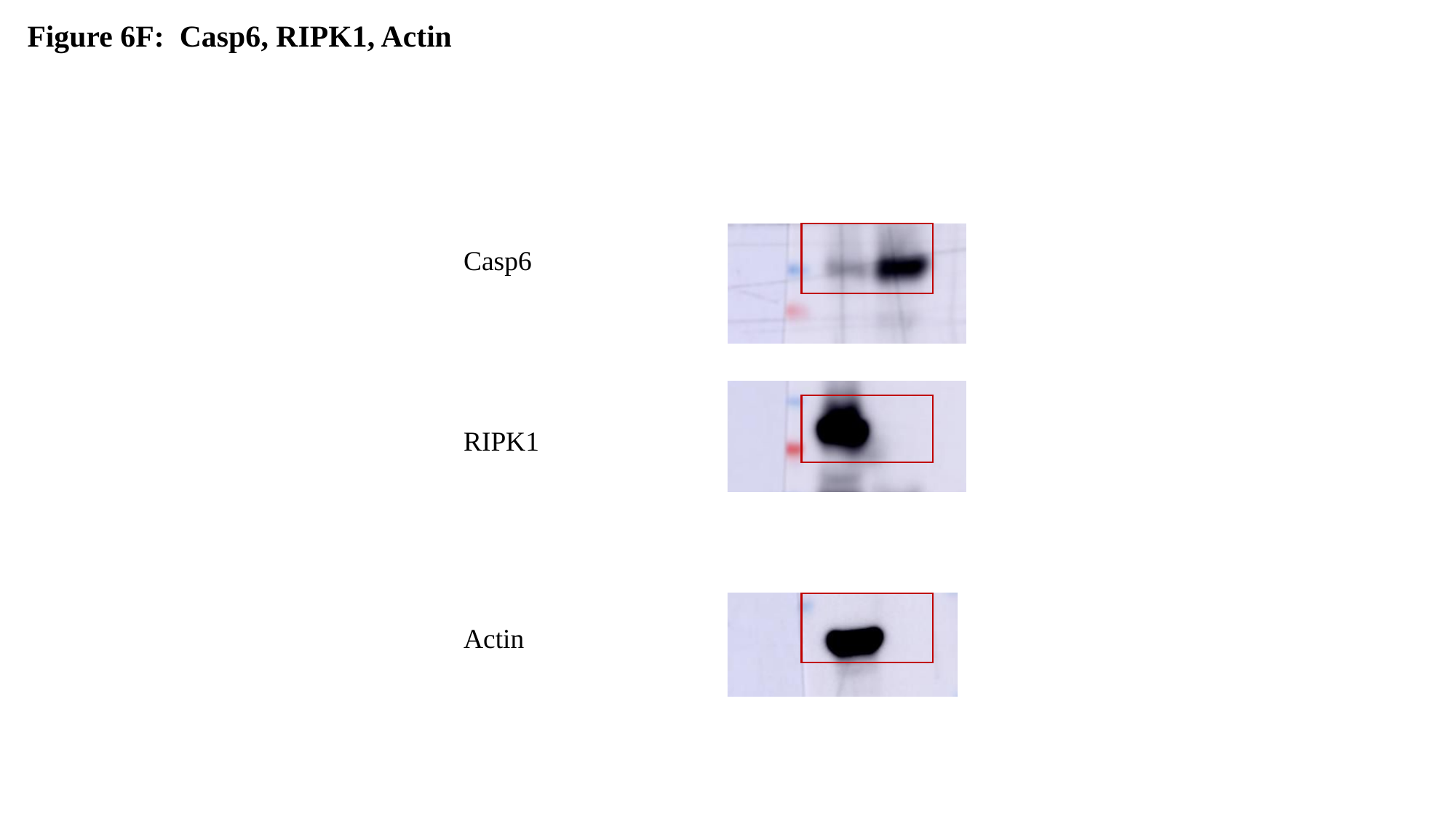

Figure 6F: Casp6, RIPK1, Actin
Casp6
RIPK1
Actin

## Slide 5
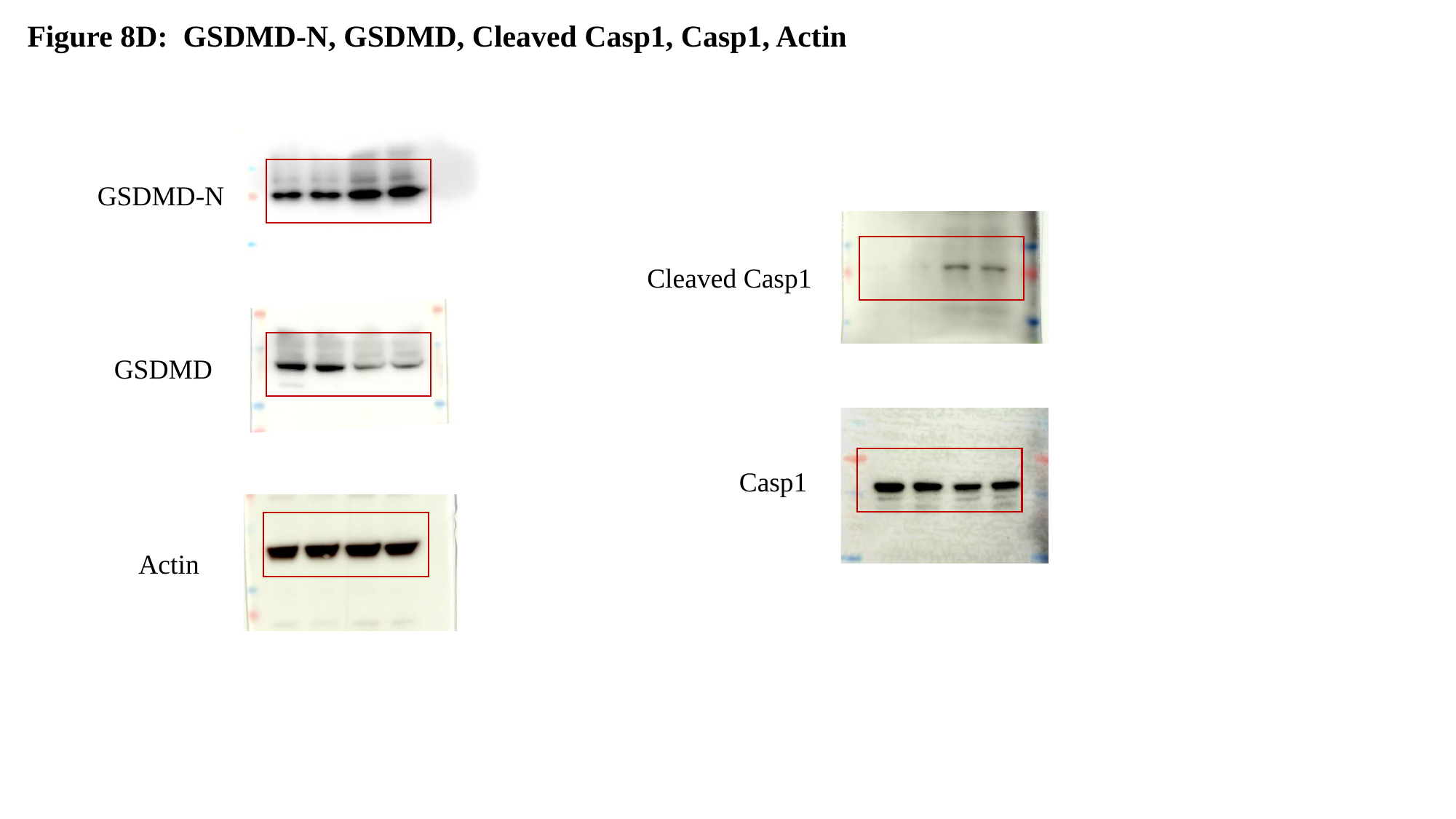

Figure 8D: GSDMD-N, GSDMD, Cleaved Casp1, Casp1, Actin
GSDMD-N
Cleaved Casp1
GSDMD
Casp1
Actin
